# GSK3β and Plk1 sequentially phosphorylate ATP–citrate lyase to promote homologous recombination

**DOI:** 10.1126/sciadv.aeg1097

**Published:** 2026-09-09

**Authors:** Shinan Zhou, Xinyu Zhou, Qinfu Chen, Xueying Yuan, Shukai Zhu, Jun Huang, Fangwei Wang, Haiyan Yan

**Affiliations:** ^1^ School of Medicine, Hangzhou City University, Hangzhou, China.; ^2^ Department of Gynecologic Oncology, Women’s Hospital, Zhejiang University School of Medicine, Hangzhou, China.; ^3^ MOE Laboratory of Biosystems Homeostasis and Protection, Life Sciences Institute, Zhejiang University, Hangzhou, China.; ^4^ Zhejiang Key Laboratory of Geriatrics and Geriatrics Institute of Zhejiang Province, Affiliated Zhejiang Hospital, Zhejiang University School of Medicine, Hangzhou, China.; ^5^ State Key Laboratory of Transvascular Implantation Devices, Hangzhou, China.

## Abstract

Accurate repair of DNA double-strand breaks (DSBs) by homologous recombination (HR) is essential for genome stability. Nuclear production of acetyl–coenzyme A (acetyl-CoA) by ATP–citrate lyase (ACLY) promotes HR, yet how ACLY is regulated during the DNA damage response (DDR) remains unclear. Here, we identify a phosphorylation-dependent signaling axis in which glycogen synthase kinase 3β (GSK3β) and Polo-like kinase 1 (Plk1) act sequentially on ACLY to facilitate HR-mediated repair of DSBs induced by ionizing radiation. Following AKT-dependent phosphorylation of ACLY at Ser^455^, GSK3β phosphorylates ACLY at Thr^447^, generating a docking site for Plk1, which in turn phosphorylates ACLY at Ser^442^. This phosphorylation cascade, enhanced by radiation, sustains histone acetylation, supports the accumulation of BRCA1 and RAD51 at DSBs, and confers cellular resistance to poly(ADP-ribose) polymerase (PARP) inhibition. Together, our findings define an AKT-GSK3β-Plk1-ACLY signaling module that links the DDR to nuclear metabolism, revealing a critical mechanism by which kinase signaling facilitates acetyl-CoA–dependent chromatin remodeling to preserve genome integrity.

## INTRODUCTION

The maintenance of genome stability relies on the faithful repair of DNA double-strand breaks (DSBs). These highly cytotoxic lesions, arising from endogenous metabolic processes or exogenous insults, represent a major source of genomic alterations and can drive tumorigenesis if repaired inaccurately (*1*).

Eukaryotic cells resolve DSBs primarily through two pathways: the error-prone nonhomologous end joining (NHEJ) pathway, which operates throughout the cell cycle, and the high-fidelity homologous recombination (HR) pathway, which is restricted to the S and G_2_ phases (*2*). The choice between these pathways is tightly regulated by chromatin context and the coordinated assembly of repair machineries (*3*). Disruption of this regulation causes genomic instability and underpins the synthetic lethality of poly(ADP-ribose) polymerase (PARP) inhibitors in HR-deficient cancers, such as those harboring mutations in *BRCA1* or *BRCA2* (*4*–*6*).

Chromatin state is a critical determinant of DSB repair pathway choice (*7*, *8*). Histone posttranslational modifications regulate chromatin accessibility and create docking platforms for repair factors (*9*). In particular, histone acetylation fosters a permissive chromatin environment for HR by relaxing nucleosome structure and facilitating the recruitment of HR mediators (*8*, *10*–*23*). Global histone acetylation is constrained by the nuclear availability of acetyl–coenzyme A (acetyl-CoA) (*24*–*26*). Adenosine 5′-triphosphate (ATP)–citrate lyase (ACLY) is a central metabolic enzyme that generates nuclear acetyl-CoA, thereby linking cellular metabolism to chromatin acetylation (*27*). Although ACLY is known to promote histone acetylation and HR (*28*), the mechanisms that regulate ACLY during the DNA damage response (DDR) remain poorly defined.

In this study, we identify a phosphorylation-dependent signaling axis that couples the DDR to ACLY-mediated control of chromatin metabolism. We demonstrate that, primed by AKT-dependent phosphorylation of ACLY, glycogen synthase kinase 3β (GSK3β) and Polo-like kinase 1 (Plk1) sequentially phosphorylate ACLY to sustain histone acetylation, ensure efficient HR, and confer cellular resistance to PARP inhibition. Our findings reveal a mechanistic link between kinase signaling, nuclear metabolism, and the maintenance of genome integrity.

## RESULTS

### Unbiased proteomics identifies ACLY as a phosphorylation-dependent Plk1 interactor

**Fig. 1.**
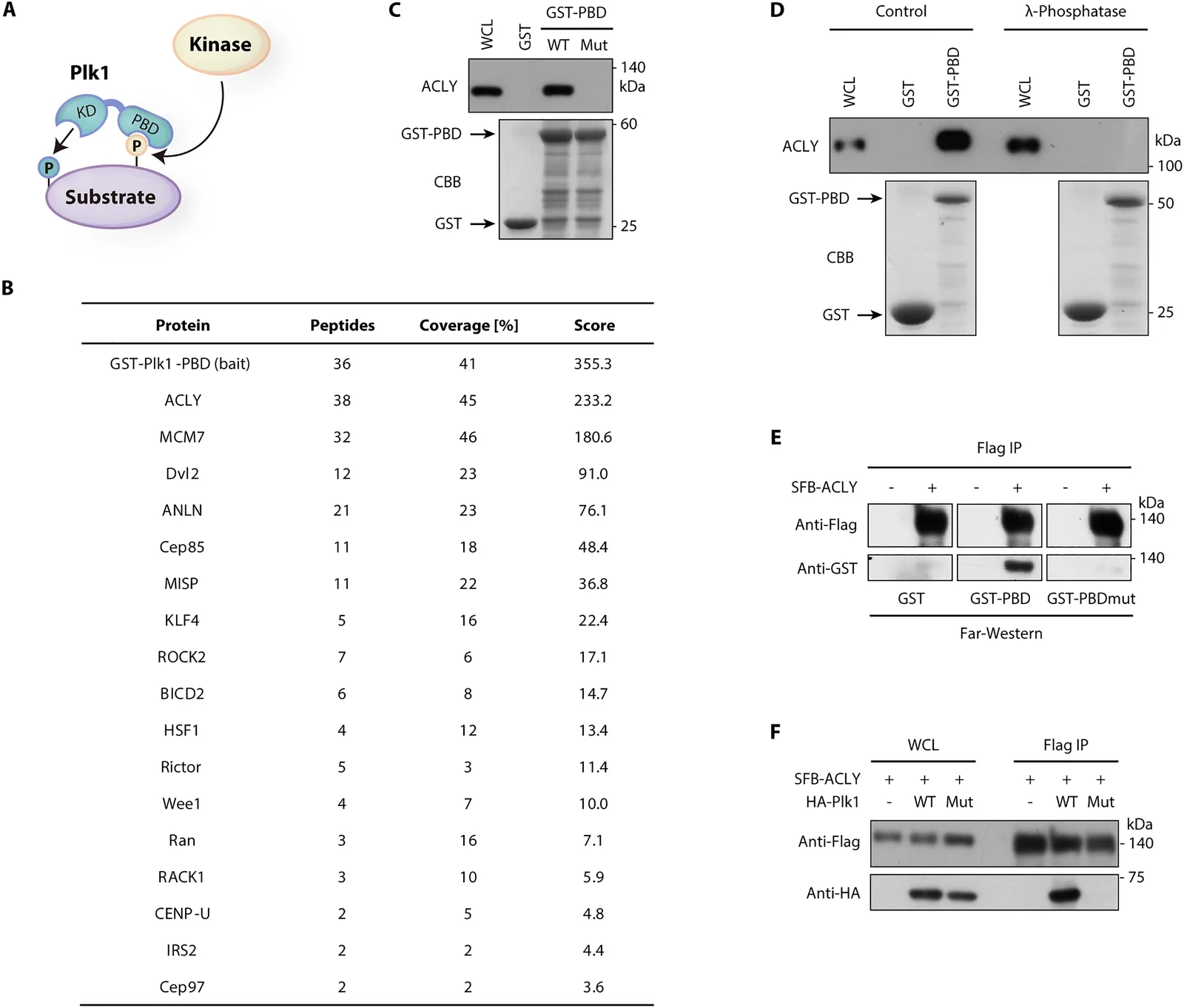
Unbiased proteomics identifies ACLY as a phosphorylation-dependent Plk1 interactor. (**A**) Schematic illustrating Plk1 substrate priming by an upstream kinase, enabling PBD recognition and subsequent phosphorylation by the Plk1 kinase domain (KD). (**B**) HeLa cells were synchronized using a double-thymidine block and harvested 7 hours after release from the second block. Lysates were subjected to pull-down with GST, GST-PBD, or a phospho-binding–deficient GST-PBD mutant, followed by mass spectrometry. Proteins specifically enriched in the GST-PBD pull-down and identified by at least two unique peptides are listed. See also data S1 to S3. (**C**) Cell lysates were subjected to pull-down with the indicated GST fusion proteins, followed by immunoblotting for ACLY. The bottom panel shows Coomassie Brilliant Blue (CBB) staining of GST fusion proteins. (**D**) Cell lysates were treated with λ-phosphatase or a vehicle control prior to pull-down with the indicated GST fusion proteins, followed by immunoblotting and CBB staining. (**E**) HEK293T cells with or without SFB-ACLY expression were subjected to IP with anti-Flag beads and analyzed by immunoblotting and Far-Western blotting using the indicated probes. (**F**) HEK293T cells coexpressing SFB-ACLY and HA-Plk1 (WT or PBDmut) were subjected to IP with anti-Flag beads, followed by immunoblotting. WCL, whole-cell lysate (C, D, and F). See also fig. S1.

Plk1 plays a critical role in cell cycle regulation, particularly during mitosis (*29*). Its unique structure consists of an N-terminal kinase domain and a C-terminal polo-box domain (PBD) (Fig. 1A). The PBD recognizes primed phospho-motifs on substrates, enabling Plk1 to phosphorylate distinct sites and function as a key signaling node (*30*, *31*). To identify Plk1 interactors that might participate in DNA repair, we performed unbiased proteomic screens in S/G_2_-phase cells, when Plk1 expression increases and HR is active (*32*, *33*).

HeLa cells were synchronized using a double-thymidine block-and-release protocol, and pull-down assays were performed with recombinant Plk1 PBD fused to glutathione *S*-transferase (GST-PBD). Control assays used GST alone or a phospho-binding–deficient GST-PBD mutant (hereafter “GST-PBDmut”) carrying H538A/K540M substitutions (*30*, *31*). Mass spectrometry identified numerous proteins that specifically bound to wild-type (WT) GST-PBD (data S1 to S3), including known Plk1 interactors such as MCM7, Dvl2, ROCK2, BICD2, and CENP-U (Fig. 1B) (*34*–*39*). Notably, ACLY emerged as a top-ranked hit.

We next biochemically validated ACLY as a direct, phosphorylation-dependent PBD binder. Endogenous ACLY was pulled down from HeLa cell lysates by GST-PBD, but not by GST or GST-PBDmut (Fig. 1C). This interaction was confirmed in human embryonic kidney (HEK) 293T cells expressing ACLY fused to an SFB tag (S tag, Flag tag, and streptavidin-binding peptide) (fig. S1). The inability of GST-PBDmut to interact with ACLY suggested a phosphorylation-dependent mechanism. Consistent with this, treatment of HeLa lysates with λ-phosphatase abolished the GST-PBD–mediated pull-down of ACLY (Fig. 1D). Far-Western analysis demonstrated a direct interaction, as SFB-ACLY immunopurified from HEK293T cells was recognized by GST-PBD, but not by GST or GST-PBDmut (Fig. 1E). Furthermore, coimmunoprecipitation (co-IP) experiments showed that SFB-ACLY associated with hemagglutinin (HA)–tagged Plk1, but not with a phospho-binding–deficient HA-Plk1 mutant (H538A/K540M) (Fig. 1F). Collectively, these results establish ACLY as a direct and phosphorylation-dependent interactor of Plk1.

### Phosphorylation of ACLY at Thr^447^ generates a docking site for Plk1

**Fig. 2.**
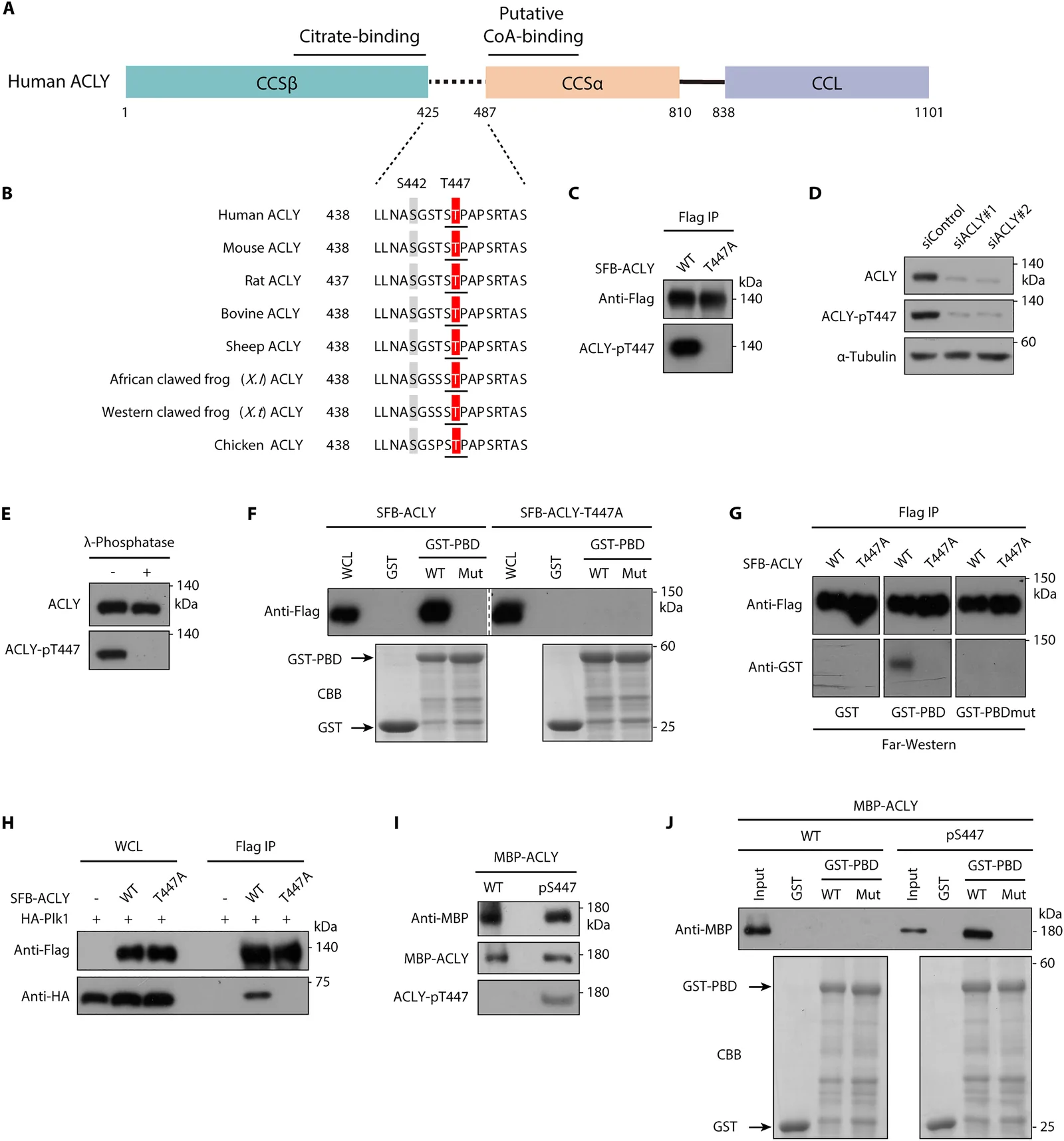
Phosphorylation of ACLY at Thr^447^ generates a docking site for Plk1. (**A**) Domain organization of human ACLY, indicating the citrate-binding and putative CoA-binding regions. Amino acid positions are numbered; the dotted line represents the linker connecting CCSβ and CCSα domains. (**B**) Sequence alignment of the ACLY linker region across vertebrates. The conserved ST^447^P motif is underlined. (**C**) HEK293T cells expressing SFB-ACLY (WT or T447A) were subjected to IP with anti-Flag beads, followed by immunoblotting. (**D**) HeLa cells were transfected with control siRNA or two distinct siRNAs targeting ACLY. Cells were harvested 48 hours later and analyzed by immunoblotting. (**E**) HeLa cell lysates were treated with λ-phosphatase or a vehicle control and analyzed by immunoblotting. (**F**) HEK293T cells expressing SFB-ACLY (WT or T447A) were subjected to pull-down with the indicated proteins, followed by immunoblotting and CBB staining. Irrelevant lanes were excised (indicated by a vertical line). (**G**) SFB-ACLY (WT or T447A) was immunoprecipitated from HEK293T cells and analyzed by immunoblotting and Far-Western blotting. (**H**) HEK293T cells coexpressing SFB-ACLY (WT or T447A) and HA-Plk1 were subjected to IP with anti-Flag beads, followed by immunoblotting. (**I**) Recombinant MBP-ACLY (WT or pS447) was analyzed by immunoblotting with the antibodies against MBP, ACLY, and ACLY-pT447. (**J**) Recombinant MBP-ACLY (WT or pS447) was subjected to pull-down with the indicated GST fusion proteins, followed by immunoblotting and CBB staining. See also fig. S2.

Human ACLY is a 1101–amino acid protein comprising an N-terminal citryl-CoA synthetase (CCS) module and a C-terminal citryl-CoA lyase (CCL) domain (Fig. 2A) (*40*, *41*). The CCS module itself contains CCSβ and CCSα subregions connected by a linker (residues 426 to 486). To map the Plk1-binding site on ACLY, we scanned its sequence for the canonical PBD docking motifs (Ser-pSer/Thr-Pro, where “p” represents phosphorylation). This analysis revealed only one Ser-Thr-Pro motif, which resides in the linker region (Fig. 2B). Thr^447^ within this conserved motif was previously reported as a phosphorylation site (*42*). Using a pan-Ser-pThr-Pro antibody, we detected a signal on immunopurified WT SFB-ACLY from asynchronously growing HEK293T cells. This signal was abolished in the SFB-ACLY-T447A mutant, where Thr^447^ was substituted with alanine (fig. S2A), confirming Thr^447^ as the phosphorylation site within this motif.

To specifically monitor Thr^447^ phosphorylation, we generated a phospho-specific antibody against ACLY-T447 phosphorylation (ACLY-pT447). This antibody recognized WT SFB-ACLY but not the T447A mutant and detected endogenous ACLY-pT447 in asynchronous HeLa cells, a signal that was markedly reduced upon ACLY depletion (Fig. 2, C and D). Furthermore, antibody recognition was abolished by λ-phosphatase treatment of cell lysates (Fig. 2E), confirming that the antibody specifically reports Thr^447^ phosphorylation in cells. Notably, ACLY-pT447 levels were comparable between asynchronous cells and those blocked in S phase by thymidine treatment (fig. S2B).

We next determined whether Thr^447^ phosphorylation is required for the ACLY-Plk1 interaction. Both pull-down and Far-Western assays revealed that GST-PBD bound WT ACLY but failed to interact with the T447A mutant (Fig. 2, F and G). Accordingly, co-IP experiments showed that SFB-ACLY, but not the T447A mutant, associated with HA-Plk1 (Fig. 2H). These data indicate that Thr^447^ phosphorylation is essential for ACLY to bind Plk1.

We then asked whether phosphorylation at Thr^447^ is sufficient to confer PBD binding. To this end, we bacterially expressed and purified maltose-binding protein (MBP)–tagged ACLY containing a phosphoserine at the equivalent position (MBP-ACLY-pS447) (Fig. 2I), using an expanded genetic code system (*43*, *44*). In pull-down assays, GST-PBD specifically bound to MBP-ACLY-pS447 but not to unmodified MBP-ACLY (Fig. 2J). Together, these results demonstrate that phosphorylation of ACLY at Thr^447^ is both necessary and sufficient to create a docking site for the PBD of Plk1.

### GSK3β phosphorylates ACLY at Thr^447^ to prime Plk1 binding

**Fig. 3.**
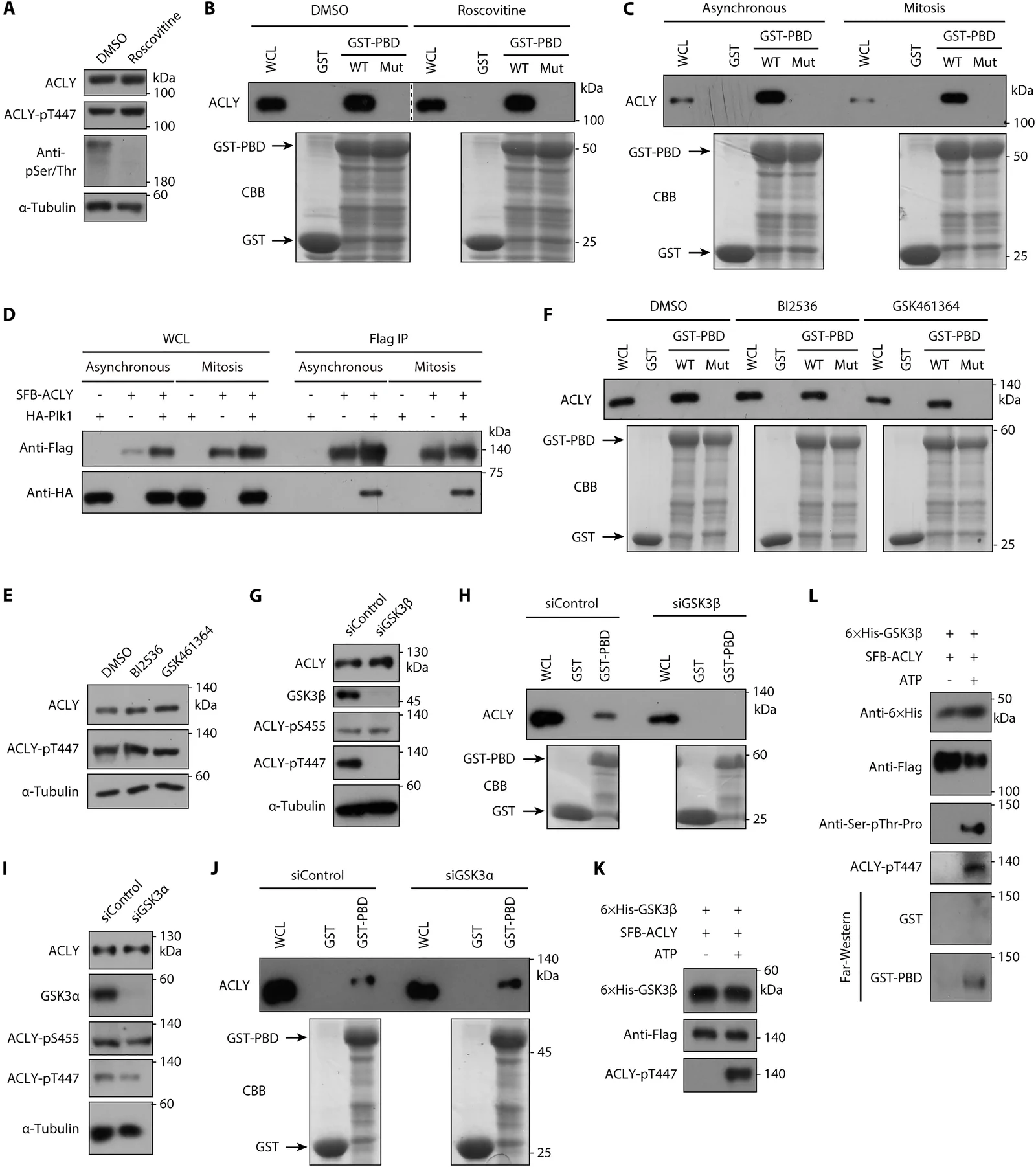
GSK3β phosphorylates ACLY at T447 to prime Plk1 binding. (**A** and **B**) HeLa cells were synchronized in G_2_ phase using a double-thymidine block. Seven hours after release, cells were treated with roscovitine or DMSO (control) for 2 hours and then either immunoblotted (A) or subjected to pull-down with the indicated GST fusion proteins, followed by immunoblotting and CBB staining (B). Irrelevant lanes were excised (indicated by a vertical line). (**C**) Asynchronous or nocodazole-arrested mitotic HeLa cells were subjected to pull-down with the indicated proteins, followed by immunoblotting and CBB staining. (**D**) Asynchronous or nocodazole-arrested mitotic HeLa cells expressing HA-Plk1 and/or SFB-ACLY were subjected to IP with anti-Flag beads, followed by immunoblotting. (**E** and **F**) HeLa cells were treated with BI2536, GSK461364, or DMSO (control) for 3 hours. Cells were then either immunoblotted (E) or subjected to pull-down with the indicated proteins, followed by immunoblotting and CBB staining (F). (**G** and **H**) HeLa cells were transfected with control or GSK3β siRNA. Forty-eight hours later, cells were either immunoblotted (G) or subjected to pull-down with the indicated proteins, followed by immunoblotting and CBB staining (H). (**I** and **J**) HeLa cells were transfected with control or GSK3α siRNA. Forty-eight hours later, cells were either immunoblotted (I) or subjected to pull-down with the indicated proteins, followed by immunoblotting and CBB staining (J). (**K**) SFB-ACLY immunoprecipitated from HEK293T cells pretreated with LY2090314 for 3 hours was subjected to in vitro phosphorylation with recombinant 6×His-GSK3β in the presence or absence of ATP, followed by immunoblotting with the antibodies against GSK3β, Flag tag, and ACLY-pT447. (**L**) SFB-ACLY was immunoprecipitated and phosphorylated as in (K) and then analyzed by immunoblotting and Far-Western blotting. See also fig. S3.

We sought to identify the upstream kinase responsible for phosphorylating ACLY at Thr^447^. Priming phosphorylation of Plk1 docking sites is often mediated by the cyclin-dependent kinase 1 (Cdk1) (*45*–*47*). The Ser-Thr^447^-Pro motif conforms to the Cdk1 consensus (pSer/Thr-Pro). However, inhibiting Cdk1 activity with roscovitine in G_2_-synchronized HeLa cells, as evidenced by loss of immunoblotting signals detected by a pan-pSer/Thr antibody, did not reduce ACLY-pT447 levels or its binding to GST-PBD (Fig. 3, A and B). Furthermore, since Cdk1 activity peaks in mitosis, we compared the ACLY-Plk1 interaction in asynchronous versus nocodazole-arrested mitotic cells. The interaction was equally robust under both conditions, as assessed by GST-PBD pull-down and co-IP (Fig. 3, C and D), arguing against Cdk1-dependent regulation. We also tested whether Plk1 itself phosphorylates its own binding site, a phenomenon known as self-priming (*37*–*39*). However, inhibition of Plk1 with the small-molecule inhibitors BI2536 or GSK461364 had minimal effect on ACLY-pT447 levels or the ACLY-PBD interaction (Fig. 3, E and F). These results indicate that neither Cdk1 nor Plk1 serves as the primary kinase for ACLY-T447.

We next examined the sequence context of Thr^447^, which resides within a conserved region (Ser-Thr^447^-Pro-Ala-Pro-Ser; see Fig. 2B) matching the canonical GSK3 consensus (Ser/Thr-*x*-*x*-*x*-pSer/Thr, where “*x*” represents any amino acid) (*48*, *49*). This prompted us to evaluate a potential role for GSK3. Using small interfering RNA (siRNA) to deplete the two main mammalian GSK3 isoforms, we found that knockdown of GSK3β, but not GSK3α, abolished ACLY-pT447 in asynchronous cells and eliminated its pull-down by GST-PBD (Fig. 3, G to J), indicating a specific requirement for GSK3β.

To determine if GSK3β directly phosphorylates Thr^447^, we performed in vitro kinase assays. Using bacterially purified, unmodified MBP-ACLY as a substrate, 6×His-GSK3β failed to generate detectable Thr^447^ phosphorylation (fig. S3), suggesting that a priming modification may be required. In contrast, when we used SFB-ACLY immunopurified from HEK293T cells pretreated with the GSK3β inhibitor LY2090314 to reduce background ACLY-T447 phosphorylation, 6×His-GSK3β phosphorylated Thr^447^ in an ATP-dependent manner (Fig. 3K). This phosphorylation enabled SFB-ACLY to bind GST-PBD in the Far-Western assay (Fig. 3L). The differential outcomes imply that the cell-derived ACLY likely contains the necessary priming modification for GSK3β-mediated phosphorylation at Thr^447^. Collectively, these results identify GSK3β as the physiologically relevant kinase that phosphorylates ACLY at Thr^447^ to generate a docking site for Plk1.

### ACLY promotes BRCA1 recruitment to DSBs without affecting Plk1 activity

Plk1 activation requires the binding of its PBD to primed phospho-motifs, an event that relieves autoinhibition of the kinase domain (*50*–*52*). We therefore examined whether ACLY influences Plk1 activity. Plk1 activity was assessed following ACLY depletion in HeLa cells synchronized in G_2_ or mitosis. siRNA-mediated knockdown of ACLY did not affect Plk1 phosphorylation at Thr^210^ (Plk1-pT210) (fig. S4, A and B), a well-established marker of its catalytic activity (*53*, *54*). In contrast, depletion of the known Plk1 activator Bora abolished Plk1-pT210, validating the assay. These findings indicate that ACLY is dispensable for Plk1 activation.

**Fig. 4.**
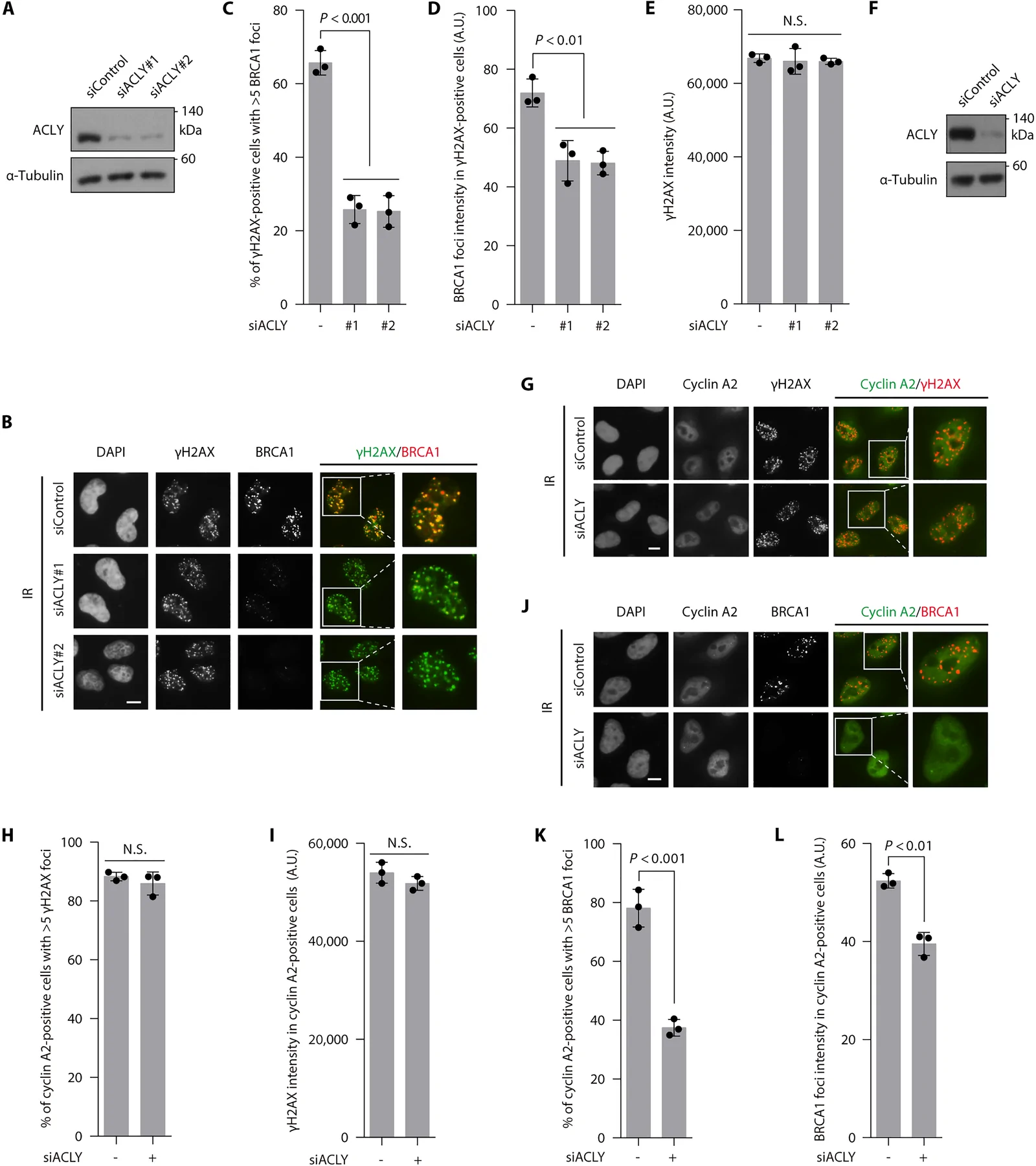
ACLY promotes BRCA1 recruitment to DSBs without affecting Plk1 activity. (**A** to **E**) HeLa cells were transfected with the indicated siRNAs. Forty-eight hours posttransfection, cells were irradiated (2 Gy) and processed for immunoblotting (A) or immunofluorescence staining 4 hours later. Representative images are shown (B). Quantification of the percentage of γH2AX-positive cells with >5 BRCA1 foci (C), BRCA1 foci intensity in γH2AX-positive cells (D), and nuclear γH2AX intensity (E) is shown. Data are presented as means ± SD from three independent experiments [*n* > 300 cells per condition for (C) and (E); *n* > 150 cells per condition for (D)]. (**F** to **I**) HeLa cells were transfected with the indicated siRNAs. Forty-eight hours posttransfection, cells were irradiated (2 Gy) and processed for immunoblotting (F) or immunofluorescence staining 4 hours later. Representative images are shown (G). Quantification of the percentage of cyclin A2–positive cells with >5 γH2AX foci (H) and nuclear γH2AX intensity in cyclin A2–positive cells (I) is shown. Data are presented as means ± SD from three independent experiments [*n* > 300 cells per condition for (H) and (I)]. (**J** to **L**) HeLa cells were transfected and irradiated as in (F) and processed for immunofluorescence staining 4 hours later. Representative images are shown (J). Quantification of the percentage of cyclin A2–positive cells with >5 BRCA1 foci (K) and BRCA1 foci intensity in cyclin A2–positive cells (L) is shown. Data are presented as means ± SD from three independent experiments [*n* > 300 cells per condition for (K); *n* > 150 cells per condition for (L)]. Scale bars, 10 μm (B, G, and J). A.U., arbitrary units (D, E, I, and L). N.S., not significant. See also fig. S4.

ACLY has been shown to facilitate efficient recruitment of BRCA1 to DSBs marked by nuclear γH2AX foci (*28*). Consistent with this previous report (*28*), immunofluorescence analysis of HeLa cells 4 hours after ionizing radiation (IR) revealed that the majority of cells contained γH2AX foci; moreover, at least half of these γH2AX-positive cells contained >5 BRCA1 foci (fig. S4C). Upon ACLY depletion with two distinct siRNAs, the proportion of γH2AX-positive cells exhibiting >5 BRCA1 foci significantly decreased from 65.8 to ∼25% (Fig. 4, A to C). Quantification of fluorescence intensity showed that ACLY depletion markedly reduced BRCA1 foci intensity in γH2AX-positive cells (Fig. 4D and fig. S4, D to F), but did not alter the intensity of nuclear γH2AX (Fig. 4E and fig. S4, G to I). This indicates that the observed defect in BRCA1 recruitment is not due to differences in DSB formation or initial signaling. Additionally, we observed a significant reduction in 4′,6-diamidino-2-phenylindole (DAPI) intensity following ACLY depletion with siRNA#2, but not with siRNA#1 (fig. S4J), suggesting possible off-target effects of siRNA#2. We therefore used siRNA#1 (referred to as siACLY) in most of the subsequent experiments.

We further quantified γH2AX and BRCA1 foci specifically in S/G_2_-phase cells, which were identified by positive immunostaining for cyclin A2 (fig. S4K). Costaining of γH2AX with cyclin A2 revealed that, at 4 hours post-IR, over 85.9% of cyclin A2–positive cells contained >5 γH2AX foci (Fig. 4, F to H), and ACLY depletion did not affect the intensity of nuclear γH2AX (Fig. 4I and fig. S4, L to N). Costaining of BRCA1 with cyclin A2 showed that ∼80% of cyclin A2–positive cells contained >5 BRCA1 foci; moreover, ACLY depletion not only significantly reduced the proportion of cyclin A2–positive cells with >5 BRCA1 foci (Fig. 4, J and K), but also markedly diminished BRCA1 foci intensity in these cells (Fig. 4L and fig. S4, O to Q). Together, these results demonstrate that ACLY facilitates BRCA1 recruitment to DSBs without affecting Plk1 activity.

### GSK3β-mediated ACLY-T447 phosphorylation is required for efficient BRCA1 recruitment to DSBs

**Fig. 5.**
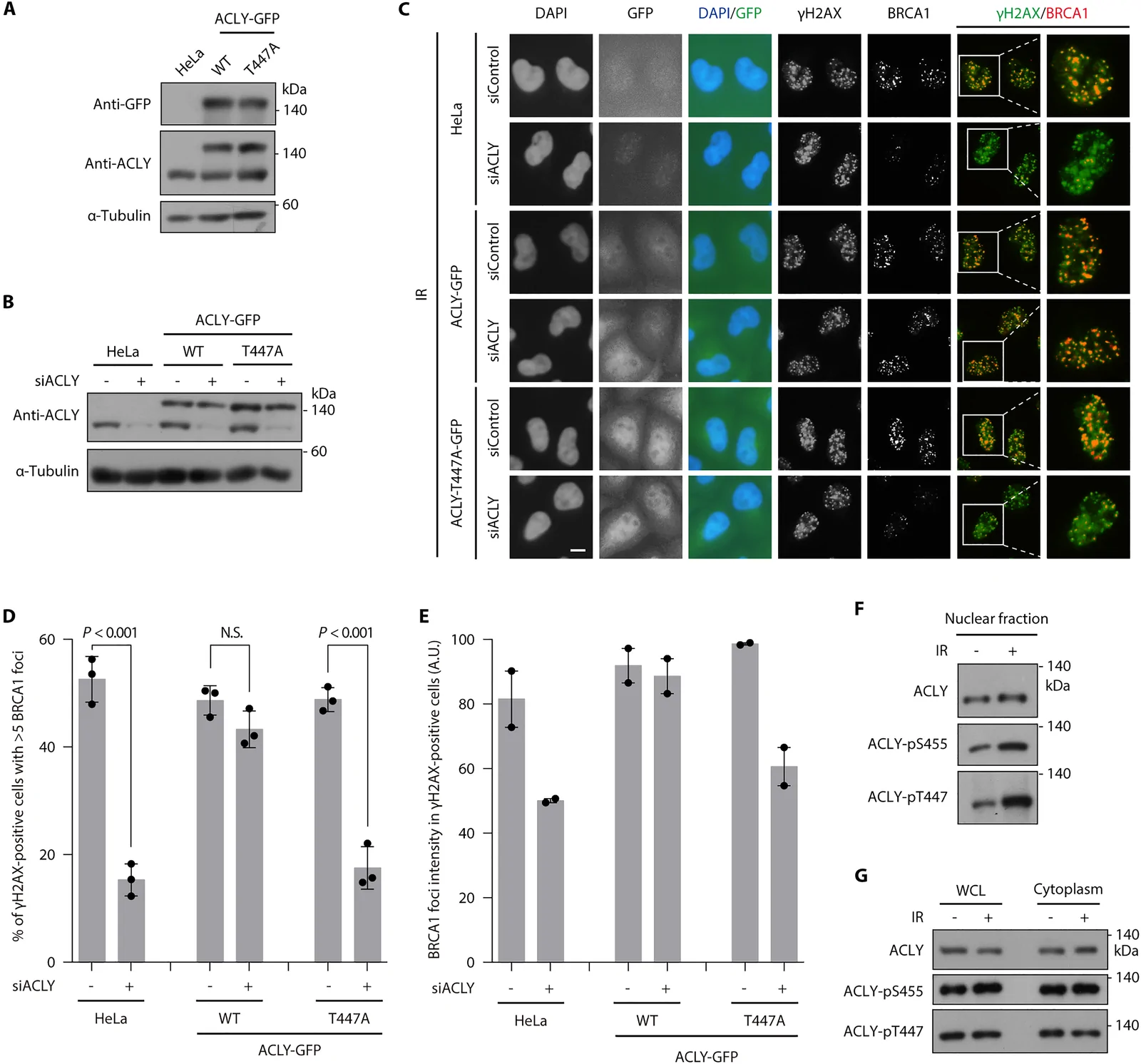
GSK3β-mediated ACLY-T447 phosphorylation is required for efficient BRCA1 recruitment to DSBs. (**A**) Immunoblot analysis of whole-cell lysates from HeLa cells stably expressing ACLY-GFP (WT or T447A). (**B** to **E**) Parental HeLa cells and HeLa cells stably expressing ACLY-GFP (WT or T447A) were transfected with control or ACLY siRNA. Two days posttransfection, cells were irradiated (2 Gy) and processed for immunoblotting (B) or immunofluorescence staining 4 hours later. Representative images are shown (C). Scale bar, 10 μm. Quantification of the percentage of γH2AX-positive cells containing >5 BRCA1 foci is presented as mean ± SD from three independent experiments (D) (*n* > 300 cells per condition). Quantification of BRCA1 foci intensity in γH2AX-positive cells is presented as mean and range from two independent experiments (E) (*n* > 100 cells per condition). (**F** and **G**) HeLa cells were irradiated (5 Gy) or left untreated. One hour later, cells were lysed for subcellular fractionation; the nuclear fraction (F) and the whole-cell lysate and cytoplasmic fraction (G) were then analyzed by immunoblotting. See also figs. S5 and S6.

Next, we investigated the functional significance of ACLY-T447 phosphorylation for BRCA1 recruitment to DSBs. To this end, we generated HeLa cell lines stably expressing siRNA-resistant ACLY–green fluorescent protein (GFP) or a phosphorylation-deficient ACLY-T447A-GFP mutant at near-endogenous levels (Fig. 5A). These cell lines enabled depletion of endogenous ACLY while maintaining expression of the respective exogenous variant (Fig. 5B). Quantification of the percentage of γH2AX-positive cells containing more than five BRCA1 foci revealed that WT ACLY-GFP rescued BRCA1 foci formation upon endogenous ACLY depletion, whereas the ACLY-T447A mutant failed to do so (Fig. 5, C and D). Quantification of fluorescence intensity showed that ACLY depletion markedly reduced BRCA1 foci intensity in γH2AX-positive cells expressing the ACLY-T447A-GFP mutant, but not in those expressing WT ACLY-GFP (Fig. 5E and fig. S5, A and B). Furthermore, costaining of BRCA1 with cyclin A2 demonstrated that ACLY depletion significantly reduced both the percentage of BRCA1 foci–positive cells and BRCA1 foci intensity in S/G_2_-phase cells expressing the ACLY-T447A-GFP mutant, but not in those expressing WT ACLY-GFP (fig. S5, C to I). These data establish that Thr^447^ phosphorylation is essential for ACLY to promote BRCA1 accumulation at DSBs.

We next examined whether DNA damage regulates ACLY-T447 phosphorylation. Notably, IR treatment enhanced ACLY-T447 phosphorylation in the nuclear fraction of HeLa cells (Fig. 5F), but not in whole-cell lysates or the cytoplasmic fraction (Fig. 5G).

Given our observation that GSK3β is the kinase responsible for phosphorylating ACLY at Thr^447^, we next determined the contribution of GSK3β activity to BRCA1 recruitment to DSBs. To this end, we established HeLa-derived cell lines stably expressing siRNA-resistant WT GSK3β-Myc or the kinase-dead mutant GSK3β-K85A-Myc (fig. S6A) (*55*). Following knockdown of endogenous GSK3β by siRNA, WT GSK3β-Myc, but not the GSK3β-K85A-Myc mutant, efficiently supported BRCA1 accumulation at DSBs (fig. S6, B to G). These data demonstrate that the kinase activity of GSK3β is required for its function in promoting BRCA1 accumulation at DSBs, consistent with its role in phosphorylating ACLY at Thr^447^.

We further examined BRCA1 recruitment in HeLa cells depleted of ACLY and/or GSK3β. While depletion of either ACLY or GSK3β alone reduced BRCA1 accumulation at DSBs, codepletion of both ACLY and GSK3β caused a further decrease in BRCA1 recruitment compared to either single knockdown alone (fig. S6, H to J). These data suggest that GSK3β has both ACLY-dependent and ACLY-independent activities in promoting BRCA1 recruitment to DSBs. The ACLY-independent activity could involve direct phosphorylation of repair factors in the DDR. Together, these results demonstrate that GSK3β-mediated phosphorylation of ACLY at Thr^447^ is required for efficient BRCA1 recruitment to DSBs, and that this phosphorylation event is enhanced in the nucleus by IR.

### Phosphorylation at Ser^442^ is required for ACLY to promote BRCA1 accumulation at DSBs

We next examined whether Plk1 regulates ACLY via phosphorylation. Plk1 preferentially phosphorylates substrates containing a consensus motif of Asp/Glu/Asn-*x*-Ser/Thr-Φ (*56*). Scanning the ACLY sequence revealed several conserved serine and threonine residues fitting this consensus, including Thr^94^, Thr^224^, Ser^442^, Ser^525^, Thr^582^, and Ser^826^. Among these, Thr^94^, Ser^442^, and Thr^582^ are documented phosphorylation sites in public databases (e.g., PhosphoSitePlus).

**Fig. 6.**
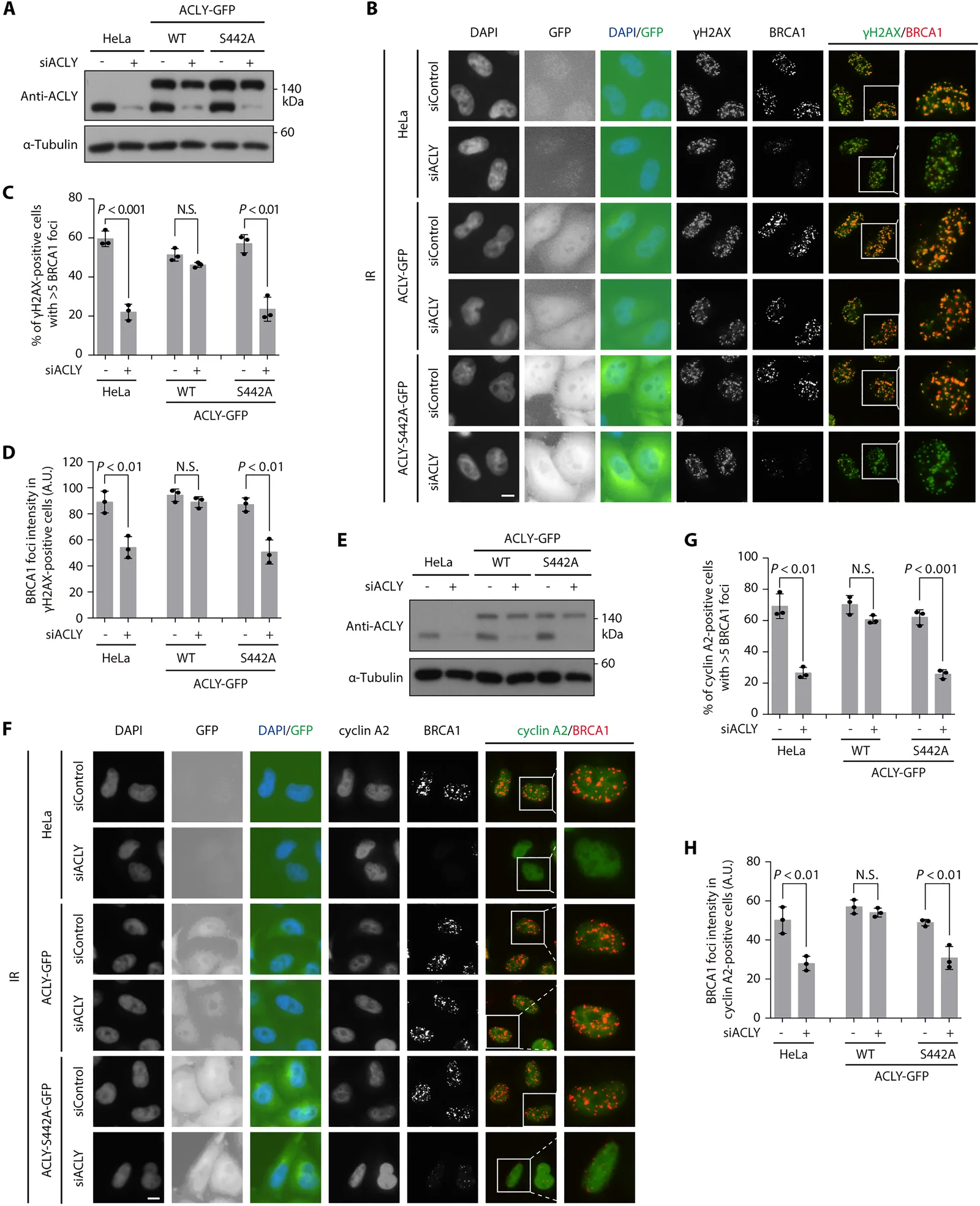
Plk1 phosphorylates ACLY-S442 to promote BRCA1 accumulation at DSBs. (**A** to **D**) Parental HeLa cells and HeLa cells stably expressing ACLY-GFP (WT or S442A) were transfected with control or ACLY siRNA. Forty-eight hours posttransfection, cells were irradiated (2 Gy) and processed for immunoblotting (A) or immunofluorescence staining 4 hours later. Representative images are shown (B). Quantification of the percentage of γH2AX-positive cells with >5 BRCA1 foci (C) and BRCA1 foci intensity in γH2AX-positive cells (D) is shown. Data are presented as means ± SD from three independent experiments [*n* > 300 cells per condition for (C); *n* > 150 cells per condition for (D)]. (**E** to **H**) Parental HeLa cells and HeLa cells stably expressing ACLY-GFP (WT or S442A) were transfected with control or ACLY siRNA. Forty-eight hours posttransfection, cells were irradiated (2 Gy) and processed for immunoblotting (E) or immunofluorescence staining 4 hours later. Representative images are shown (F). Quantification of the percentage of cyclin A2–positive cells with >5 BRCA1 foci (G) and BRCA1 foci intensity in cyclin A2–positive cells (H) is shown. Data are presented as means ± SD from three independent experiments [*n* > 300 cells per condition for (G); *n* > 150 cells per condition for (H)]. Scale bars, 10 μm (B and F). See also figs. S7 and S8.

To identify functionally relevant phosphorylation sites, we generated HeLa cell lines stably expressing siRNA-resistant ACLY-GFP mutants. Following endogenous ACLY depletion and IR treatment, a pentuple-alanine mutant (ACLY-5A-GFP; T94A/T224A/S525A/T582A/S826A) supported BRCA1 recruitment to DSBs (fig. S7, A to C), whereas the ACLY-S442A-GFP mutant failed to do so (Fig. 6, A to C).

We confirmed that the ACLY-S442A mutation did not affect the intensity of nuclear γH2AX (fig. S7, D to F), but significantly reduced BRCA1 foci intensity in γH2AX-positive cells (Fig. 6D and fig. S7, G to I). Furthermore, the S442A mutation abolished the ability of ACLY-GFP to promote BRCA1 foci formation in cyclin A2–positive cells (Fig. 6, E to H, and fig. S7, J to L).

We further established HeLa-derived cell lines stably expressing the ACLY-S442D-GFP mutant in which Ser^442^ was mutated to aspartate. Following depletion of endogenous ACLY, this mutant did not rescue BRCA1 foci formation compared to the WT protein (fig. S7, M to O), indicating that the S442D mutation does not functionally mimic Ser^442^ phosphorylation.

We next developed a phospho-specific antibody against ACLY Ser^442^ phosphorylation (ACLY-pS442). This antibody recognized endogenous ACLY immunopurified from HeLa cells and specifically detected WT ACLY-GFP, but not the ACLY-S442A mutant (fig. S7, P and Q). The signal was abolished by λ-phosphatase treatment of cell lysates (fig. S7R), confirming antibody specificity.

The DNA repair factors BRCA1 and 53BP1 are thought to act antagonistically to promote HR and NHEJ, respectively (*2*). As previously reported (*28*), ACLY knockdown increased the intensity of 53BP1 foci in HeLa cells exhibiting γH2AX foci (fig. S8, A to F). Moreover, 53BP1 foci formation was elevated in HeLa cells in which endogenous ACLY was replaced with exogenously expressed ACLY-T447A-GFP or ACLY-S442A-GFP, but not with WT ACLY-GFP (fig. S8, G to K). Together, these results indicate that phosphorylation of ACLY at Ser^442^ is required for efficient accumulation of BRCA1 at DSBs.

### Plk1 phosphorylates ACLY at Ser^442^ to promote BRCA1 accumulation at DSBs

**Fig. 7.**
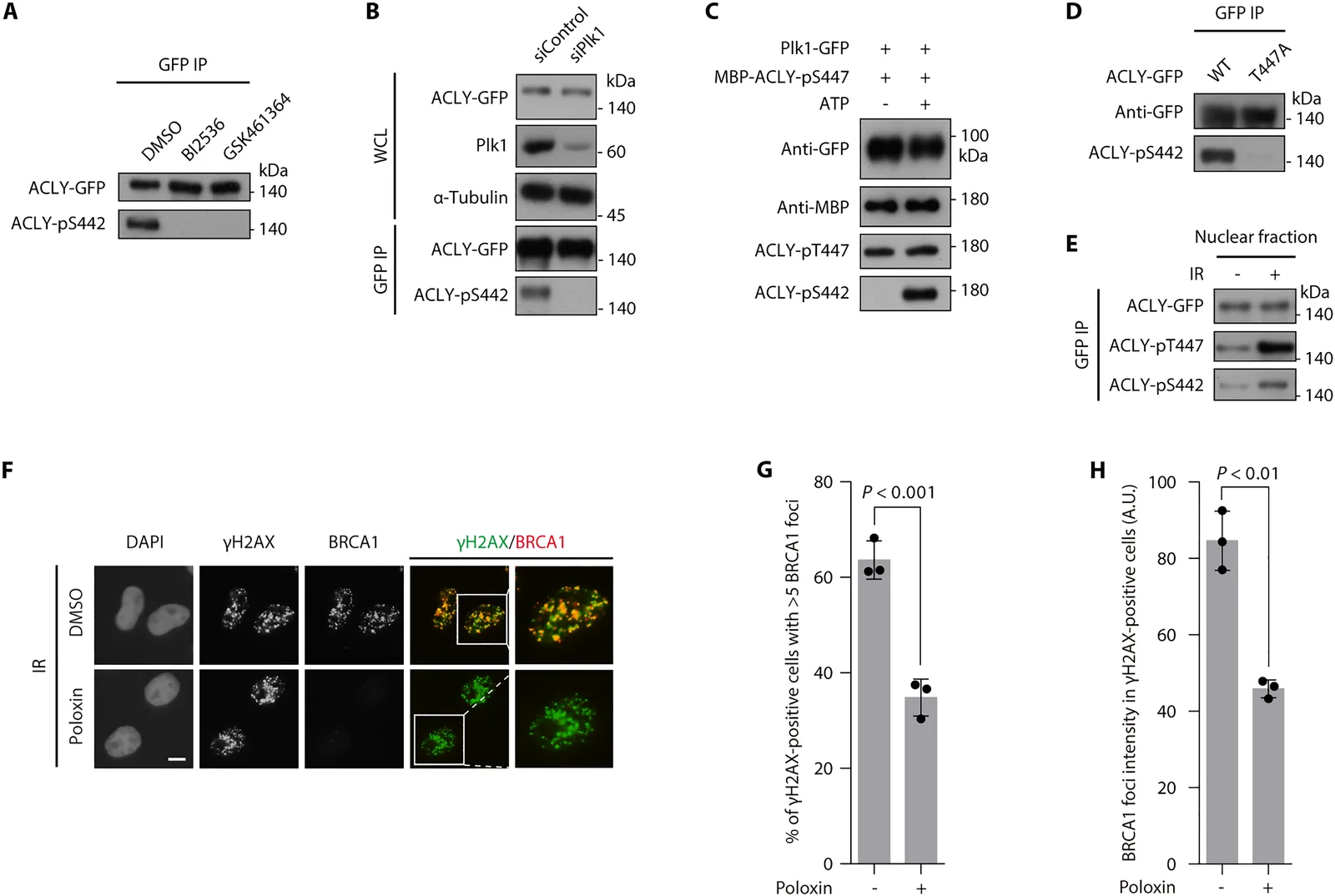
Plk1 phosphorylates ACLY at Ser^442^ to promote BRCA1 accumulation at DSBs. (**A**) HeLa cells stably expressing ACLY-GFP were treated with BI2536, GSK461364, or DMSO (control) for 3 hours. Lysates were then subjected to IP with anti-GFP beads and immunoblotting with the antibodies against GFP and ACLY-pS442. (**B**) HeLa cells stably expressing ACLY-GFP were transfected with control or Plk1 siRNA. Thirty-two hours later, cells were treated with nocodazole for 16 hours, and mitotic cells were collected for IP with anti-GFP beads and immunoblotting with the antibodies against GFP, Plk1, ACLY-pS442, and α-Tubulin. (**C**) Plk1-GFP immunopurified with anti-GFP beads from HEK293T cells was used in an in vitro kinase assay with recombinant MBP-ACLY-pS447 as a substrate. (**D**) HEK293T cells expressing ACLY-GFP (WT or T447A) were subjected to IP with anti-GFP beads, followed by immunoblotting. (**E**) HeLa cells were irradiated (10 Gy) or left untreated. Nuclear extracts were subjected to IP using anti-GFP beads and analyzed by immunoblotting 1 hour postirradiation. (**F** to **H**) HeLa cells treated with poloxin for 3 hours were irradiated (2 Gy) and processed for immunofluorescence staining 4 hours later. Representative images are shown (F). Scale bar, 10 μm. Quantification of the percentage of γH2AX-positive cells with >5 BRCA1 foci (G) and BRCA1 foci intensity in γH2AX-positive cells (H) is shown. Data are presented as means ± SD from three independent experiments [*n* > 300 cells per condition for (G); *n* > 150 cells per condition for (H)]. See also fig. S9.

We next investigated whether Plk1 catalyzes the phosphorylation of ACLY at Ser^442^. Pharmacological inhibition of Plk1 with BI2536 or GSK461364 abolished ACLY Ser^442^ phosphorylation in HeLa cells stably expressing ACLY-GFP (Fig. 7A). Similarly, Plk1 depletion by siRNA eliminated ACLY-pS442 (Fig. 7B). In vitro kinase assays further showed that immunopurified Plk1-GFP directly phosphorylated Ser^442^ on the recombinant MBP-ACLY-pS447 substrate (Fig. 7C). Moreover, ACLY Ser^442^ phosphorylation was detected on WT ACLY-GFP but not on the Plk1 docking-deficient ACLY-T447A-GFP mutant (Fig. 7D), establishing that Plk1 must first dock on phospho-Thr^447^ to phosphorylate Ser^442^. Additionally, IR exposure increased the phosphorylation of both Ser^442^ and Thr^447^ on nuclear ACLY in HeLa cells (Fig. 7E).

Poloxin is a small-molecule inhibitor that binds to the PBD of Plk1, disrupting its interaction with substrate proteins (*57*). Treatment of HeLa cells with poloxin compromised IR-induced recruitment of BRCA1 to γH2AX foci (Fig. 7, F to H, and fig. S9, A to C). These data provide strong pharmacological evidence that Plk1-PBD binding to the phospho-Thr^447^ docking site is required for BRCA1 recruitment to DSBs. In summary, these findings demonstrate that Plk1 binding to phosphorylated Thr^447^ enables subsequent phosphorylation of ACLY at Ser^442^, which is critical for promoting BRCA1 accumulation at DSBs.

### AKT-dependent phosphorylation of ACLY at Ser^455^ primes Thr^447^ phosphorylation by GSK3β

**Fig. 8.**
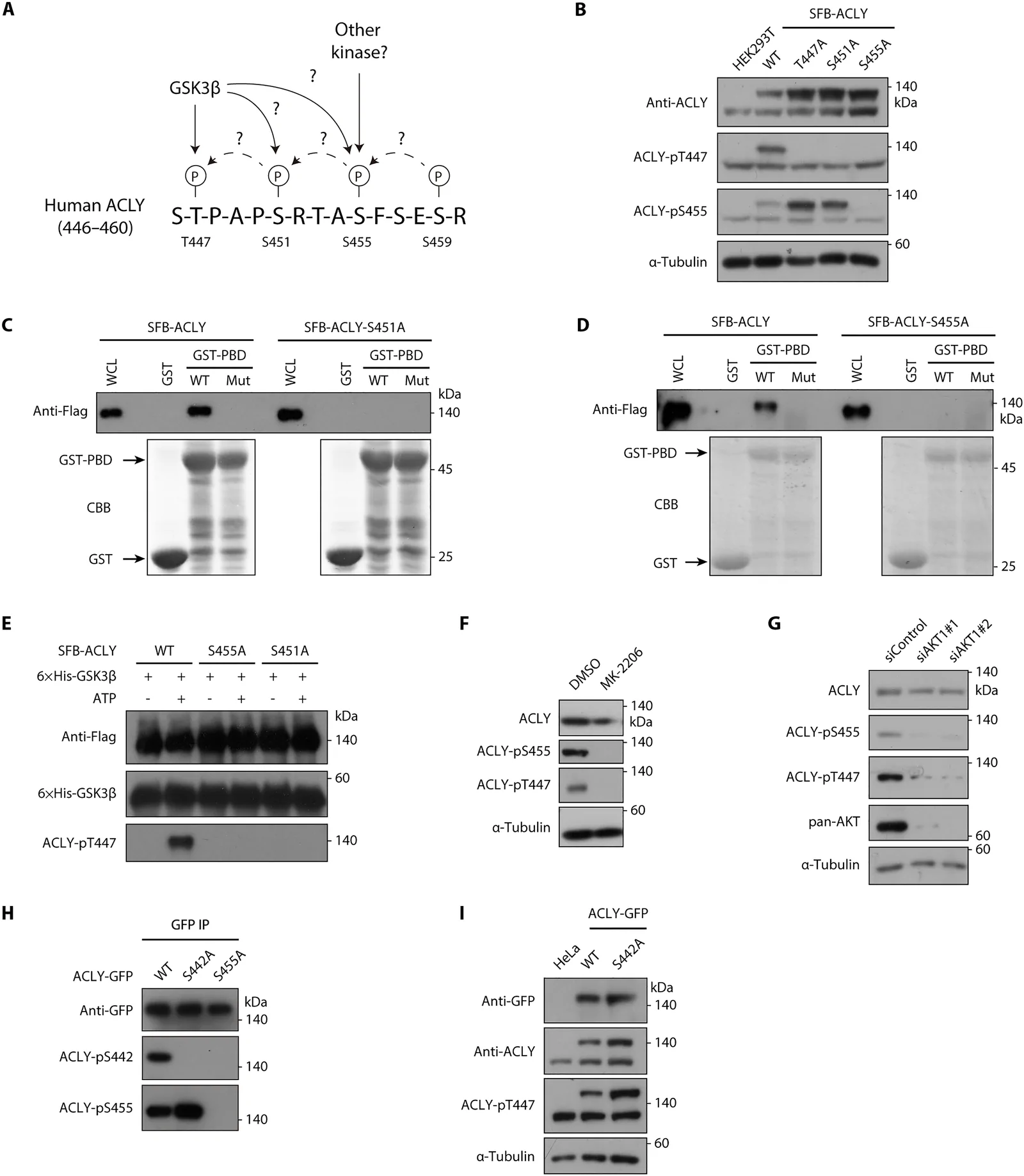
AKT-dependent phosphorylation of ACLY at Ser^455^ primes Thr^447^ phosphorylation by GSK3β. (**A**) Schematic of ACLY residues Ser^451^, Ser^455^, and Ser^459^ as potential priming sites for GSK3β-mediated phosphorylation of Thr^447^. Dashed arrows denote possible priming effect. (**B**) HEK293T cells expressing SFB-ACLY (WT, T447A, S451A, or S455A), or an empty vector control, were analyzed by immunoblotting. (**C** and **D**) HEK293T cells expressing the indicated ACLY proteins were subjected to pull-down with the indicated GST fusion proteins, followed by immunoblotting for SFB-ACLY (WT or S451A) (C) and SFB-ACLY (WT or S455A) (D) and CBB staining. (**E**) SFB-ACLY (WT, S455A, or S451A) immunoprecipitated from HEK293T cells pretreated with LY2090314 for 3 hours was subjected to in vitro phosphorylation with recombinant 6×His-GSK3β, followed by immunoblotting. (**F**) HeLa cells were treated with DMSO (control) or MK-2206 for 3 hours and analyzed by immunoblotting. (**G**) HeLa cells were transfected with control siRNA or two distinct siRNAs targeting AKT1. Cells were harvested 48 hours later and analyzed by immunoblotting. (**H**) HEK293T cells expressing ACLY-GFP (WT, S442A, or S455A) were subjected to IP with anti-GFP beads, followed by immunoblotting. (**I**) Immunoblot analysis of whole-cell lysates from parental HeLa cells and HeLa cells stably expressing ACLY-GFP (WT or S442A). See also fig. S10.

GSK3 typically requires priming phosphorylation at a residue located four residues C-terminal to its target site (*49*). Because the sequence downstream of the GSK3β target Thr^447^ contains several known phosphorylation sites (Ser^451^, Ser^455^, and Ser^459^) (Fig. 8A) (*58*, *59*), we investigated their potential as priming sites. Mutating Ser^451^ or Ser^455^ to alanine (S451A or S455A) abolished Thr^447^ phosphorylation on SFB-ACLY expressed in HEK293T cells, whereas the S459A mutation had no effect (Fig. 8B and fig. S10A). Consistently, the S451A or S455A mutations, but not the S459A mutation, disrupted the interaction between SFB-ACLY and GST-PBD (Fig. 8, C and D, and fig. S10B). Furthermore, in vitro kinase assays using immunopurified SFB-ACLY as a substrate demonstrated that 6×His-GSK3β phosphorylated Thr^447^ on WT ACLY, but not on the S451A or S455A mutants (Fig. 8E). These data establish Ser^451^ and Ser^455^ as priming sites for Thr^447^ phosphorylation by GSK3β.

Although Ser^451^ is a predicted GSK3 targeting site based on sequence consensus, we could not confirm this experimentally due to the lack of a phospho-specific antibody. We therefore sought to determine the kinase responsible for ACLY Ser^455^ phosphorylation (ACLY-pS455). Immunoblotting analysis showed that ACLY-pS455 was not reduced by mutations at Thr^447^, Ser^451^, or Ser^459^ (see Fig. 8B and fig. S10A). Although the sequence surrounding Ser^455^ fits the GSK3 consensus, Ser^455^ phosphorylation was not reduced upon depletion of GSK3β or GSK3α (see Fig. 3, G and I). The Ser^455^ context also partially matches the AKT consensus motif (Arg-*x*-Arg-*x*-*x*-Ser/Thr-Φ, where Φ is a hydrophobic amino acid) (*60*). Moreover, AKT has been shown to phosphorylate ACLY at Ser^455^ in vitro (*58*). We found that phosphorylation of ACLY at Ser^455^ was abrogated in HeLa cells treated with the AKT inhibitor MK-2206 and was substantially diminished upon siRNA-mediated knockdown of AKT1 (Fig. 8, F and G). Notably, these AKT perturbations also reduced Thr^447^ phosphorylation, positioning AKT upstream of GSK3β in the phosphorylation of ACLY-T447. Furthermore, consistent with ACLY Thr^447^ phosphorylation being upstream of Ser^442^ phosphorylation, the ACLY-pS442–specific antibody failed to recognize the ACLY-S455A-GFP mutant immunopurified from HEK293T cells (Fig. 8H). Additionally, the S442A mutation did not affect phosphorylation of Ser^455^ or Thr^447^ on ACLY-GFP expressed in cells (Fig. 8, H and I). Thus, while Ser^442^ phosphorylation is downstream of, and dependent on, Ser^455^/Thr^447^ phosphorylation, Ser^442^ does not feedback to affect the phosphorylation of upstream sites.

Phosphorylation of ACLY at Ser^455^ has been shown to promote BRCA1 recruitment to DSBs (*28*). We next tested AKT inhibition in HeLa cells in which endogenous ACLY was replaced by the T447A or S442A mutants. Consistent with the previous report (*28*), inhibition of AKT activity by MK-2206 compromised BRCA1 accumulation at DSBs in cells expressing ACLY-GFP (fig. S10, C to E). MK-2206 treatment did not further reduce BRCA1 recruitment in cells expressing the T447A or S442A mutants of ACLY-GFP. These data indicate that AKT acts upstream of GSK3β and Plk1 in this signaling cascade and that the effect of AKT inhibition is mediated through the T447 and S442 phosphorylation sites. In summary, these results demonstrate that phosphorylation of ACLY at Ser^455^ is AKT-dependent and that this event is required to prime subsequent phosphorylation of Thr^447^ by GSK3β.

### Plk1-mediated ACLY-S442 phosphorylation promotes RAD51 loading at DSBs and confers resistance to PARP inhibition

**Fig. 9.**
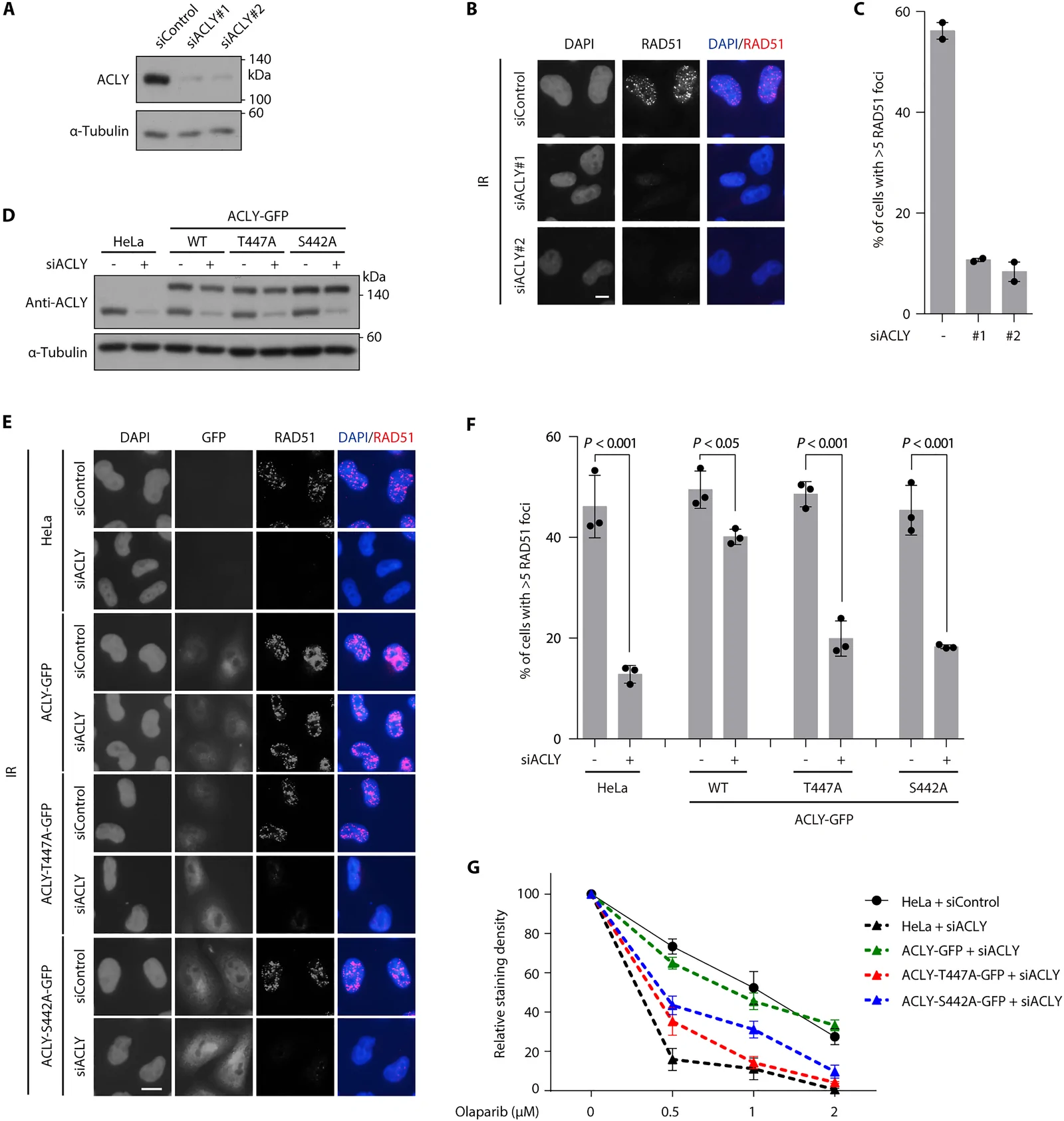
Plk1-mediated ACLY-S442 phosphorylation promotes RAD51 loading at DSBs and confers resistance to PARP inhibition. (**A** to **C**) HeLa cells were transfected with the indicated siRNAs. Forty-eight hours posttransfection, cells were irradiated (4 Gy) and processed for immunoblotting (A) or immunofluorescence staining 4 hours later. Representative images are shown (B). Quantification of the percentage of cells with >5 RAD51 foci is shown (C). Data are presented as means and range from two independent experiments (*n* > 200 cells per condition). (**D** to **F**) Parental HeLa cells and HeLa cells stably expressing ACLY-GFP (WT, T447A, or S442A) were transfected with control or ACLY siRNA. Forty-eight hours later, cells were irradiated (4 Gy) and processed for immunoblotting (D) or immunofluorescence staining 4 hours later. Representative images are shown (E). Quantification of the percentage of cells with >5 RAD51 foci is shown (F). Data are presented as means ± SD from three independent experiments (*n* > 300 cells per condition). (**G**) The indicated cell lines were transfected with control or ACLY siRNA. Two days posttransfection, cells were treated with the indicated concentrations of olaparib and subjected to clonogenic survival assays. Colonies were stained with crystal violet after 9 days. Surviving fractions were quantified from three independent experiments. Representative colony images are shown in fig. S11H. Scale bars, 10 μm (B and E). See also fig. S11.

The recruitment of BRCA1 to DSBs facilitates DNA end resection, generating single-stranded DNA (ssDNA) overhangs that are essential for initiating HR (*61*). RAD51 is then loaded onto this ssDNA to catalyze the homology search and strand invasion steps of accurate repair (*62*). Given our finding that the Plk1-ACLY axis is required for BRCA1 recruitment, we next asked whether it also promotes the downstream loading of RAD51. Immunofluorescence analysis 4 hours after IR revealed RAD51 foci in ∼50% of HeLa cells (fig. S11A). Consistent with the defect in BRCA1 recruitment, ACLY depletion markedly reduced the proportion of cells exhibiting >5 RAD51 foci from 56.5% to 8.0–10.5% (Fig. 9, A to C). Crucially, RAD51 foci formation was restored in cells expressing WT ACLY-GFP following endogenous ACLY depletion, but not in cells expressing ACLY-T447A-GFP or ACLY-S442A-GFP (Fig. 9, D to F). These data indicate that Plk1-mediated phosphorylation of ACLY at Ser^442^ is essential for efficient RAD51 loading at DSBs.

Costaining of RAD51 with cyclin A2 further demonstrated that endogenous ACLY depletion significantly reduced RAD51 foci count in cyclin A2–positive HeLa cells and stable cell lines expressing the T447A or S442A mutants, but not in those expressing WT ACLY-GFP (fig. S11, B to G). These analyses indicate that the defect in RAD51 loading upon expression of the phosphorylation-deficient ACLY mutants is specific to S/G_2_-phase cells.

Because PARP inhibitors are synthetically lethal with HR defects (*63*), we next tested whether Plk1-mediated ACLY phosphorylation confers resistance to these agents. Clonogenic survival assays were performed using cells treated with the PARP inhibitor olaparib following depletion of endogenous ACLY and reconstitution with siRNA-resistant ACLY-GFP, ACLY-T447A-GFP, or ACLY-S442A-GFP. Control HeLa cells showed intrinsic resistance to olaparib at concentrations up to 2.0 μM, which was abrogated by ACLY depletion (Fig. 9G and fig. S11H). Resistance to olaparib was restored by exogenous expression of WT ACLY-GFP in ACLY-depleted cells, but not by the T447A or S442A mutants. Together, these results demonstrate that the Plk1-ACLY signaling axis, through phosphorylation of Ser^442^, is critical for promoting RAD51 loading during HR and for conferring cellular resistance to PARP inhibition.

### Plk1-mediated phosphorylation of ACLY-S442 enhances the ACLY-Tip60 interaction and sustains histone H4 acetylation

**Fig. 10.**
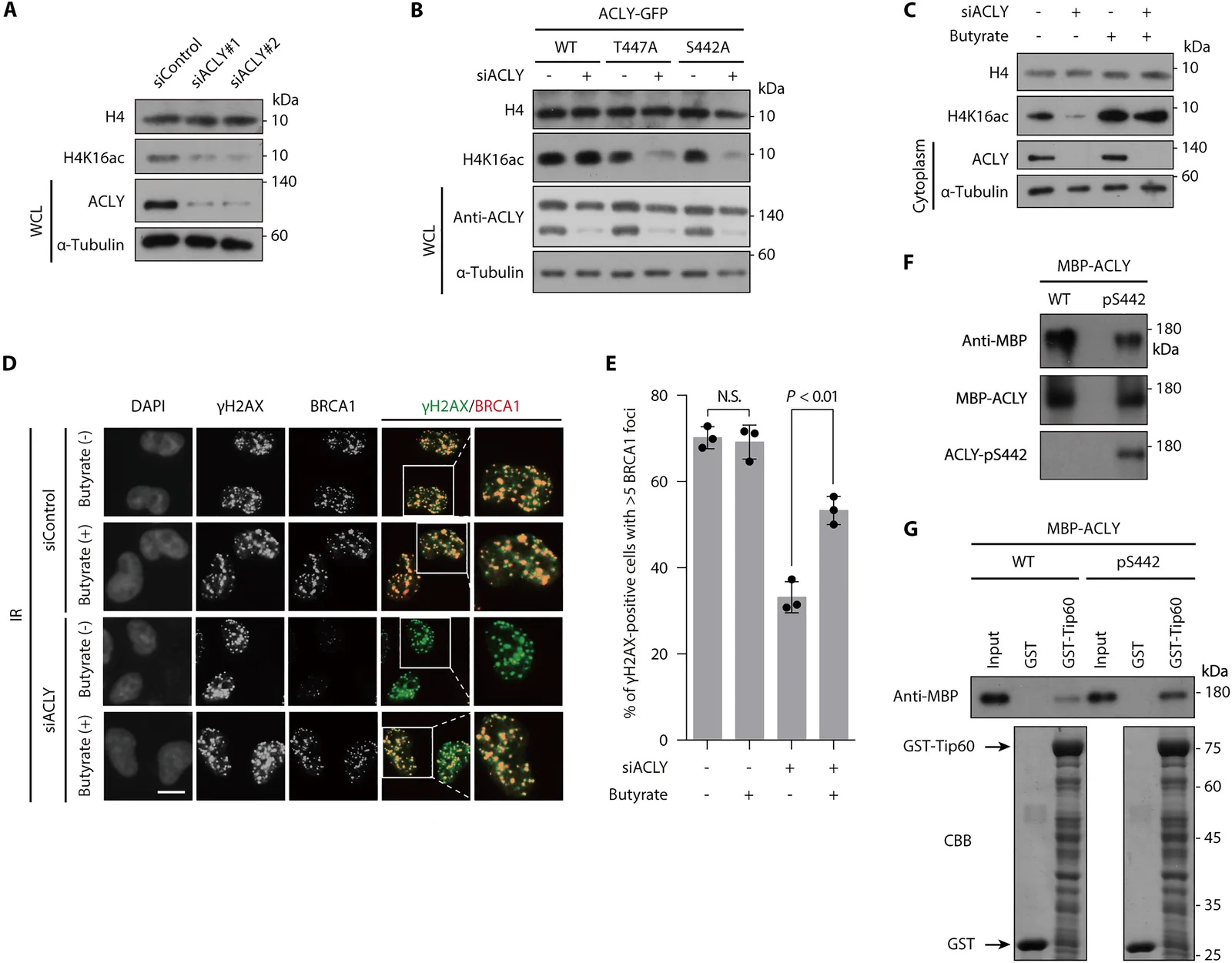
Plk1-mediated phosphorylation of ACLY-S442 enhances the ACLY-Tip60 interaction and sustains histone H4 acetylation. (**A**) HeLa cells were transfected with the indicated siRNAs. Forty-eight hours later, whole-cell lysates and acid-extracted histones were prepared and analyzed by immunoblotting. (**B**) HeLa cells stably expressing ACLY-GFP (WT, T447A, or S442A) were transfected with control or ACLY siRNA. Forty-eight hours later, whole-cell lysates and acid-extracted histones were immunoblotted. (**C**) HeLa cells were transfected with control or ACLY siRNA. Forty-eight hours later, cells were treated with water (control) or butyrate for 5 hours. Immunoblots of acid-extracted histones and the cytoplasmic fraction are shown. (**D** and **E**) HeLa cells were transfected with control or ACLY siRNA. Forty-eight hours later, cells were treated with water (control) or butyrate for 5 hours, irradiated (2 Gy), and, after 4-hour recovery, processed for immunofluorescence staining. Representative images are shown (D). Scale bar, 10 μm. Quantifications of the percentage of γH2AX-positive cells containing >5 BRCA1 foci is shown (E). Data are presented as means ± SD from three independent experiments (*n* > 300 cells per condition). (**F**) Recombinant MBP-ACLY (WT or pS442) was analyzed by immunoblotting with the antibodies against MBP, ACLY, and ACLY-pS442. (**G**) Recombinant MBP-ACLY (WT or pS442) was subjected to pull-down with GST-Tip60 or GST alone, followed by immunoblotting and CBB staining. See also fig. S12.

Histone acetylation, including that of H4 at Lys^16^, is dynamically regulated in response to DNA damage (*17*, *64*). H4 Lys^16^ acetylation (H4K16ac) promotes a chromatin environment permissive for HR by relaxing internucleosomal contacts and antagonizing the recruitment of 53BP1 (*16*, *65*–*72*). Nuclear acetyl-CoA produced by ACLY serves as an essential cofactor for histone acetylation (*27*, *28*). We therefore asked whether Plk1-dependent phosphorylation of ACLY at Ser^442^ contributes to the maintenance of H4K16ac. Consistent with prior findings (*27*), knockdown of ACLY in HeLa cells using two independent siRNAs markedly reduced H4K16ac levels (Fig. 10A). Reconstitution of endogenous ACLY-depleted cells with WT ACLY-GFP restored H4K16ac to normal levels, whereas expression of phosphorylation-defective mutants (T447A or S442A) failed to restore acetylation (Fig. 10B). These data indicate that Plk1-mediated phosphorylation of ACLY at Ser^442^ is required to sustain H4K16 acetylation.

Since H4K16ac facilitates HR by promoting chromatin openness and inhibiting 53BP1 recruitment (*16*, *17*), we next tested whether the HR defect caused by ACLY depletion could be rescued by restoring histone acetylation. Treatment of ACLY-depleted cells with the histone deacetylase (HDAC) inhibitor butyrate not only restored H4K16ac levels, but also enabled BRCA1 foci formation at IR-induced DSBs (Fig. 10, C to E). These results support a model in which ACLY promotes HR primarily by maintaining an acetylated, HR-permissive chromatin state (fig. S12A).

The histone acetyltransferase Tip60 is known to catalyze H4K16ac and is essential for efficient HR (*17*). Given the defect of the ACLY-S442A mutant in maintaining H4K16ac, we investigated whether ACLY interacts with Tip60 and whether this interaction is modulated by Ser^442^ phosphorylation. Using an expanded genetic code system, we purified bacterially expressed MBP- ACLY containing a phosphoserine at residue 442 (MBP-ACLY-pS442) (Fig. 10F). Pull-down assays revealed a direct interaction between recombinant GST-Tip60 and MBP-ACLY, but not with GST alone. Notably, GST-Tip60 exhibited stronger binding to MBP-ACLY-pS442 than to unmodified MBP-ACLY (Fig. 10G). This indicates that phosphorylation of ACLY at Ser^442^ enhances its direct interaction with Tip60. We further bacterially expressed and purified the N-terminal fragment of 53BP1 (residues 1 to 699) fused to GST (*73*) and found that it did not bind to either MBP-ACLY or MBP-ACLY-pS442 (fig. S12B), suggesting that the effect of ACLY on 53BP1 foci formation is indirect. Together, these findings establish that Plk1-catalyzed phosphorylation of ACLY at Ser^442^ strengthens its binding to Tip60, maintains histone H4 acetylation, and supports efficient HR.

## DISCUSSION

Our study defines a phosphorylation-dependent signaling axis that integrates the DDR with nuclear metabolism to promote HR (fig. S12A). We demonstrate that, upon priming phosphorylation of ACLY by AKT, GSK3β and Plk1 act sequentially on ACLY to sustain histone acetylation and promote accurate DSB repair. This ordered kinase cascade, enhanced by DNA damage signaling and propagated through AKT, GSK3β, and Plk1, directly couples DDR activation to the metabolic production of nuclear acetyl-CoA, revealing a critical mechanism for shaping the chromatin landscape required for efficient HR.

A central finding is the three-step phosphorylation relay on ACLY. AKT phosphorylates Ser^455^, priming subsequent GSK3β-dependent phosphorylation at Thr^447^, which creates a docking site for Plk1. Plk1 then binds to this primed site and phosphorylates Ser^442^. This sequential modification is essential for ACLY’s ability to support the recruitment of BRCA1 and RAD51 to DSBs and to maintain histone H4 acetylation, particularly at Lys^16^. The requirement for Plk1 docking provides a built-in checkpoint, ensuring that ACLY’s chromatin-modifying function is activated only upon receipt of appropriate upstream signals. Notably, this places Plk1, a kinase best known for its mitotic roles (*29*), and GSK3β, a key regulator of cellular signaling (*49*), in a noncanonical position as coordinators of metabolic signaling during the DDR.

A paradox arises from AKT, a well-established inhibitor of GSK3β (*49*, *74*), simultaneously acting as its priming kinase. Our data support a model of substrate-specific regulation (*75*): Localized AKT activity at sites of damage or within specific subcellular compartments provides the priming phosphorylation on ACLY, allowing an active pool of GSK3β to engage its primed substrate. This context-dependent cooperation between ostensibly antagonistic kinases affords precise spatiotemporal control over ACLY activation.

Functionally, the principal output of the GSK3β-Plk1-ACLY axis is the maintenance of histone acetylation that supports HR. The rescue of BRCA1 recruitment in ACLY-depleted cells by HDAC inhibition underscores that sustaining acetylation is at least one of the key mechanisms. Furthermore, we show that phosphorylation at Ser^442^ strengthens the direct interaction between ACLY and the histone acetyltransferase Tip60. This finding supports a model of metabolite channeling, whereby ACLY locally supplies acetyl-CoA to acetyltransferases at chromatin, forming a chromatin-associated “metabolon” that enables rapid, targeted acetylation at DSBs (*76*–*79*).

Therapeutically, this signaling-metabolism interface has important implications. The GSK3β-Plk1-ACLY axis acts as a tunable regulator of HR proficiency. Tumors with hyperactive signaling through this pathway may acquire resistance to PARP inhibitors, while its inhibition could sensitize HR-proficient cancers to these agents (*40*, *80*). The existence of cancer-associated ACLY mutations at critical phospho-sites (e.g., T447M and S442I) warrants investigation into their impact on PARP inhibitor sensitivity. Thus, our findings nominate this axis and its components as promising therapeutic targets.

Several questions remain for future investigation. Structural studies are needed to elucidate how phosphorylation at Ser^442^ modulates ACLY’s association with binding partners. Prior work suggests that the linker region (residues 426 to 486) containing Ser^442^ may exert autoinhibitory effects (*41*); testing whether Plk1-mediated phosphorylation relieves such intramolecular inhibition will be an important next step. Furthermore, defining the genomic loci most influenced by this pathway and evaluating its clinical relevance in vivo will be essential for understanding its broader physiological and pathological significance.

In summary, we uncover a fundamental regulatory principle whereby the DDR directly engages nuclear metabolism through a dedicated phosphorylation cascade. By licensing ACLY to fuel histone acetylation, this pathway ensures that the metabolic state of the cell is harnessed to enforce accurate DNA repair. Our work provides a paradigm for the convergence of kinase signaling and nuclear metabolism in maintaining genome stability, with direct implications for cancer therapy.

## MATERIALS AND METHODS

### Cell lines, plasmids, siRNAs, transfection, and drug treatments

All cell lines were obtained from the American Type Culture Collection (ATCC) and cultured in Dulbecco’s modified Eagle’s medium (DMEM) supplemented with 10% fetal bovine serum (FBS; Gibco) and 1% penicillin/streptomycin at 37°C in a humidified incubator with 5% CO_2_. HeLa cells stably expressing ACLY-GFP (WT, T447A, S442A, or 5A) or GSK3β-Myc (WT or K85A) were selected and maintained in medium containing blasticidin (2 μg/ml; Sigma-Aldrich).

The SFB-ACLY construct was generated by transferring ACLY cDNA from an entry vector into a Gateway-compatible destination vector containing an N-terminal triple tag SFB. The pBos-ACLY-GFP and pBos-Plk1-GFP constructs were generated by replacing the H2B coding sequence in pBos-H2B-GFP (BD Pharmingen) with ACLY and Plk1 cDNAs, respectively, each flanked by Kpn I/Bam HI restriction sites. The pBos-GSK3β-Myc construct was created by replacing the H2B coding sequence in pBos-H2B-GFP (BD Pharmingen) with GSK3β cDNA flanked by Kpn I/Bam HI restriction sites, followed by replacement of GFP with a Myc tag. The MBP-ACLY-10×His construct was generated by subcloning sequences encoding ACLY and a 10×His tag into the Bam HI/Eco RI sites of pGEX-4T1 (GE Healthcare), followed by replacement of GST with MBP. The plasmid expressing 6×His-GSK3β was constructed by subcloning polymerase chain reaction (PCR)–amplified products encoding GSK3β into the pET45b vector. To construct plasmids expressing GST-Tip60 and GST-53BP1-N, cDNAs encoding human Tip60 and the N-terminal region of 53BP1 (residues 1 to 699) were amplified by PCR and subcloned into pGEX-4T1 (GE Healthcare), respectively. Plasmids encoding HA-tagged full-length Plk1 (pCS2-HA-Plk1), pCS2-HA-Plk1 PBDmut (H538A/K540M), GST-PBD (residues 326 to 603), and GST-PBDmut (H538A/K540M) were gifts from H. Yu (University of Texas Southwestern Medical Center). Point mutations were introduced using the QuikChange II XL Site-Directed Mutagenesis Kit (Agilent Technologies) or the MultiF Seamless Assembly Mix Kit (ABclonal Biotechnology). All constructs were verified by DNA sequencing.

The following siRNA duplexes were purchased from Integrated DNA Technologies (IDT) or RiboBio: siACLY#1 (5′-GCCAGAACUUGGUAGUCAAdTdT-3′), siACLY#2 (5′-GGCAUGUCCAACGAGCUCAAdTdT-3′), siGSK3α (5′-GUGAUUGGCAAUGGCUCAUdTdT-3′), siGSK3β (5′-GUAAUCCACCUCUGGCUACdTdT-3′), siPlk1 (5′-GAUCACCCUCCUUAAAUAUdTdT-3′), siBora (5′-CUAUGAGACUUCAGAUGUAdTdT-3′), siAKT1#1 (5′-GAAGGAAGUCAUCGUGGCCAA-3′), and siAKT1#2 (5′-GAGUUUGAGUACCUGAAGCUGUU-3′). siACLY#1 was used to knock down ACLY unless otherwise stated.

Transfections of siRNA were performed twice at a 24-hour interval using Oligofectamine (Invitrogen) or Lipofectamine RNAiMAX (Thermo Fisher Scientific). Plasmid transfections were carried out using FuGENE HD (Promega) or Lipofectamine 2000 (Thermo Fisher Scientific).

For cell cycle synchronization, a double thymidine block-and-release assay was used with 2 mM thymidine (Sigma-Aldrich). To induce DNA damage, cells were exposed to IR at 2, 4, 5, or 10 Gy (Rad Source). The following drugs were used: olaparib (0.5 to 2 μM), nocodazole (100 ng/ml), dimethyl sulfoxide (DMSO; 1:2000 dilution), roscovitine (100 μM), BI2536 (100 nM), GSK461364 (400 nM), LY2090314 (100 nM), poloxin (30 μM), MK-2206 (3 to 5 μM), and butyrate (5 mM), all obtained from Sigma-Aldrich or Selleck Chemicals.

### Antibodies

Primary antibodies used were as follows: ACLY (Abcam, ab40793), Flag tag (GenScript, A01868; for immunoblotting), GST (Sigma-Aldrich, G7781), HA tag (BioLegend, 16B12), Myc-tag (05-724, Millipore), MBP (New England BioLabs, E8032), pan-pSer/Thr (ABclonal, AP0893), pan-Ser-pThr-Pro (Cell Signaling Technology, D73F6; 5243), GSK3α (ABclonal, A19060), GSK3β (ABclonal, A6164), ACLY-pS455 (Cell Signaling Technology, 4331), BRCA1 (Santa Cruz Biotechnology, sc-6954), γH2AX (Abcam, ab81299), cyclin A2 (ABclonal, A19036), cyclin A2 (Proteintech, 66391-1-Ig), 53BP1 (made in-house), pan-AKT (ABclonal, A18120), RAD51 (made in-house), histone H4 (Sigma-Aldrich, 07-108), H4K16ac (Abcam, ab109463), α-tubulin (ABclonal, AC012), GFP (Abmart, M20004), Plk1 (Abcam, ab17057), and Plk1 phospho-T210 (BD Transduction, 558400). Rabbit polyclonal antibodies against ACLY phosphorylated at Thr^447^ (ACLY-pT447) and Ser^442^ (ACLY-pS442) were generated by immunizing rabbits with the synthetic phosphopeptides SGSTS(pT)PAPSRT-Cys and CNFLLNA(pS)GSTSTPA, respectively (where pS is phosphoserine and pT is phosphothreonine).

Secondary antibodies for immunoblotting were as follows: goat anti-rabbit immunoglobulin G (IgG)–horseradish peroxidase (HRP) (Cell Signaling Technology, 7074) and horse anti-mouse IgG-HRP (Cell Signaling Technology, 7076). Secondary antibodies for immunofluorescence were as follows: donkey anti-rabbit IgG–Alexa Fluor Cy3 (Jackson ImmunoResearch, 711-165-152), donkey anti-mouse IgG–Alexa Fluor Cy3 (Jackson ImmunoResearch, 715-165-150), donkey anti-rabbit IgG–Alexa Fluor 488 (Jackson ImmunoResearch, 711-545-152), donkey anti-mouse IgG–Alexa Fluor 488 (Thermo Fisher Scientific, A-21202), goat anti-rabbit IgG–Alexa Fluor 647 (Thermo Fisher Scientific, A-21244), and donkey anti-mouse IgG–Alexa Fluor Cy5 (Jackson ImmunoResearch, 715-175-151).

### Immunofluorescence microscopy

HeLa cells grown on coverslips were fixed with 2% paraformaldehyde (PFA) in phosphate-buffered saline (PBS) for 10 min at room temperature and permeabilized with 0.5% Triton X-100 in PBS for 5 min. Cells were blocked with 3% bovine serum albumin (BSA) in PBS and incubated with primary antibodies for 1 to 2 hours, followed by secondary antibodies for 1 hour, all at room temperature in 3% BSA/PBS. DNA was counterstained with DAPI for 5 min. Images were acquired using a Nikon ECLIPSE Ni microscope equipped with a Plan Apo Fluor 60× oil immersion objective (NA 1.4) and a Clara CCD camera (Andor Technology).

### Recombinant protein expression and purification

Plasmids encoding GST-, MBP-10×His–, or 6×His-tagged proteins were transformed into *Escherichia coli* BL21 (DE3) competent cells. Protein expression was induced with 0.4 mM isopropyl-β-d-thiogalactopyranoside (IPTG) at 16°C for 16 hours when the culture OD_600_ (optical density at 600 nm) reached 0.6 to 0.8. Cells were lysed by sonication in either buffer A (50 mM tris-HCl, pH 8.0, 300 mM NaCl, 1 mM EDTA, and 1% Triton X-100; for GST fusions) or buffer B (50 mM tris-HCl, pH 7.5, 300 mM NaCl, and 0.5% Triton X-100; for His-tagged fusions) or buffer C (PBS; for GST-53BP1-N). Clarified lysates were incubated with Glutathione Sepharose 4B beads (GE Healthcare) or Ni-NTA beads (Smart-Lifesciences). Beads were washed with the corresponding lysis buffer, and proteins were eluted with 100 mM glutathione or 300 mM imidazole, respectively.

For site-specific incorporation of phosphoserine, plasmids encoding MBP-ACLY-pS447-10×His and MBP-ACLY-pS442-10×His were generated by mutating the Thr^447^ codon and Ser^442^ codon, respectively, to “TAG.” Each resulting plasmid was cotransformed with the pEVOL-SEP plasmid (encoding SEP-tRNA and SEP synthetase) into C321ΔSerB cells (a derivative of C321.ΔA.exp. cells, Addgene, no. 49018), with the serB gene replaced by a kanamycin resistance cassette. Protein expression was induced with 0.4 mM IPTG at 16°C for 16 hours in the presence of 0.08% glucose and 0.2% arabinose. MBP-ACLY-pS447-10×His and MBP-ACLY-pS442-10×His were purified from sonicated lysates using a Ni-NTA column (Smart-Lifesciences) and eluted with 300 mM imidazole.

### Pull-down assays, immunoprecipitation, Far-Western blotting, phosphatase treatment, subcellular fractionation, and immunoblotting

For pull-down and immunoprecipitation (IP) assays, cells were lysed in P150 buffer (50 mM tris-HCl, pH 7.5, 150 mM NaCl, 1% Triton X-100, 10 mM MgCl_2_, and 5 mM EDTA) supplemented with 1 mM dithiothreitol, protease inhibitor cocktail (Sigma-Aldrich, P8340), 1 mM phenylmethylsulfonyl fluoride, 0.1 μM okadaic acid (Calbiochem), 10 mM NaF, 20 mM β-glycerophosphate, and benzonase (GenScript). Insoluble materials in the lysates were removed by high-speed centrifugation. For GST pull-downs, lysates were precleared with glutathione Sepharose 4B beads (GE Healthcare) and then incubated with bead-immobilized GST fusion proteins for 4 hours at 4°C. For IP, lysates were precleared with rProtein A/G beads (Smart-Lifesciences, SA032100) and then incubated with Anti-Flag M2 Affinity Gel (Sigma-Aldrich, A2220), Anti-GFP Affinity Beads (Smart-Lifesciences, SA070005), or control rProtein A/G beads for 4 hours at 4°C. For endogenous protein IP, lysates were first incubated with ACLY or Plk1 antibodies for 3 hours before adding rProtein A/G beads for an additional hour. Beads were washed three to five times with lysis buffer, and bound proteins were eluted by boiling in SDS sample buffer for immunoblotting. To carry out Far-Western blotting, the immunoprecipitated SFB-ACLY was resolved by SDS–polyacrylamide gel electrophoresis (SDS-PAGE), transferred to polyvinylidene difluoride membrane, and incubated with GST or GST-PBD (4 μg/ml; WT or PBDmut) in PBS-Tween containing 5% skimmed milk at 4°C for 16 hours, followed by immunoblotting with anti-GST antibody.

For phosphatase treatment, whole-cell lysates were incubated with λ-phosphatase (New England Biolabs) for 30 min at 30°C. For subcellular fractionation, cells were washed once with ice-cold PBS and resuspended in lysis buffer (10 mM Hepes, pH 7.4, 10 mM KCl, 1.5 mM MgCl_2_, 0.5 mM EDTA, and 0.5 mM EGTA) supplemented with 0.1% NP-40, a protease inhibitor cocktail, okadaic acid, and NaF. Following a 15-min incubation on ice, nuclear and cytoplasmic fractions were separated by centrifugation at 1000*g* for 5 min at 4°C. The nuclear pellet was washed twice with lysis buffer without NP-40 and then boiled in SDS sample buffer. SDS-PAGE and immunoblotting were performed according to standard procedures.

### In vitro kinase assays

Kinase assays were performed in a 50-μl reaction volume containing kinase reaction buffer (50 mM tris-HCl, pH 8.0, 150 mM NaCl, and 10 mM MgCl_2_) and 0.3 mM ATP, incubated at 30°C for 30 to 60 min. For assays with recombinant kinases, 0.5 μg of MBP-ACLY-10×His substrate was incubated with 0.2 μg of recombinant 6×His-GSK3β. For assays with immunoprecipitated kinases, anti-GFP immunoprecipitates from HEK293T cells expressing Plk1-GFP (lysed in P500 buffer: 50 mM tris-HCl, pH 7.5, 500 mM NaCl, 1% Triton X-100, 10 mM MgCl_2_, and 5 mM EDTA with inhibitors of protease and phosphatase) were incubated with 0.5 μg of MBP-ACLY-pS447-10×His. For assays with immunoprecipitated substrates, anti-Flag immunoprecipitates of SFB-ACLY (WT, S451A, or S455A) from HEK293T cells pretreated with LY2090314 for 3 hours were incubated with 0.2 μg of 6×His-GSK3β. Reactions were terminated by adding SDS sample buffer and boiling, followed by immunoblotting.

### Clonogenic survival assay

Parental HeLa cells or HeLa cell lines stably expressing siRNA-resistant ACLY-GFP, ACLY-S442A-GFP, and ACLY-T447A-GFP were transfected with either control, nontargeting siRNA or siRNA targeting endogenous ACLY to achieve specific depletion. Forty-eight hours posttransfection, cells were seeded into 6-cm dishes and treated with the PARP inhibitor olaparib (0.5 to 2.0 μM) or a DMSO vehicle control. The cells were then allowed to form colonies for 9 days. Subsequently, colonies were fixed with methanol and stained with 0.5% (w/v) crystal violet in 25% methanol. The staining intensity, which corresponds to the number of viable colonies, was quantified using ImageJ software. The surviving fraction for each condition was calculated by normalizing the staining density to that of the corresponding DMSO-treated control.

### Mass spectrometry

Proteins binding to GST, GST-PBD, or GST-PBDmut were separated by SDS-PAGE. Gel bands were excised, destained, and subjected to in-gel digestion with trypsin (Promega) overnight at 37°C. Peptides were desalted using StageTips and analyzed on a Q Exactive HF-X mass spectrometer (Thermo Fisher Scientific) coupled to an Easy-nLC 1200 system. Peptides were separated on a C18 column using a linear acetonitrile gradient. MS data were acquired in a data-dependent top-20 method. Raw files were processed using Proteome Discoverer software and searched against the UniProtKB database.

### Quantification, statistical analysis, and sequence alignment

Images were first acquired by focusing on DAPI staining or on γH2AX/cyclin A2 staining in a semiblinded manner (i.e., fields were selected without reference to the criteria to be scored: BRCA1, γH2AX, 53BP1, or RAD51). Within each experiment, all images were captured at the same time using identical exposure settings for each channel. Images were then exported to ImageJ. Each image was converted to 8-bit format, for quantification of DAPI or γH2AX intensity, the threshold was adjusted on the first image to clearly visualize the nuclei, and this threshold was applied to all subsequent images within the same experiment. Individual nuclear intensity values from each image were recorded in an Excel file, and the average nuclear intensity was calculated and plotted. For quantification of foci intensity or number (e.g., BRCA1, 53BP1, or RAD51), the threshold was adjusted on the first image to visualize all foci and then applied uniformly to all subsequent images within the same experiment. Foci intensity was measured specifically in cells that were also positive for γH2AX or cyclin A2.

Statistical analyses were performed using a two-tailed unpaired Student’s *t* test or Mann-Whitney *U* test in GraphPad Prism 8. A *P* value of < 0.05 was considered statistically significant. Multiple sequence alignment of ACLY orthologs was performed using UniProt (www.uniprot.org), and the resulting figures were formatted in Microsoft Word.
