# Supplementary material for: GSK3β and Plk1 sequentially phosphorylate ATP–citrate lyase to promote homologous recombination
Source: Sci Adv. 2026 Sep 9;12(37):eaeg1097. doi: 10.1126/sciadv.aeg1097 (PMC13557067; doi:10.1126/sciadv.aeg1097)
Supplement: Supplementary file 1 [file sciadv.aeg1097_sm.pdf]

Science Advances
AAAS

Supplementary Materials for

GSK3β and Plk1 sequentially phosphorylate ATP–citrate lyase to promote homologous recombination

Shinan Zhou *et al.*

Corresponding author: Jun Huang, jhuang@zju.edu.cn; Fangwei Wang, fwwang@zju.edu.cn; Haiyan Yan, yanhy@hzcu.edu.cn

Sci. Adv. **12**, eaeg1097 (2026)
DOI: 10.1126/sciadv.aeg1097

The PDF file includes:

Figs. S1 to S12
Legends for data S1 to S3

Other Supplementary Material for this manuscript includes the following:

Data S1 to S3

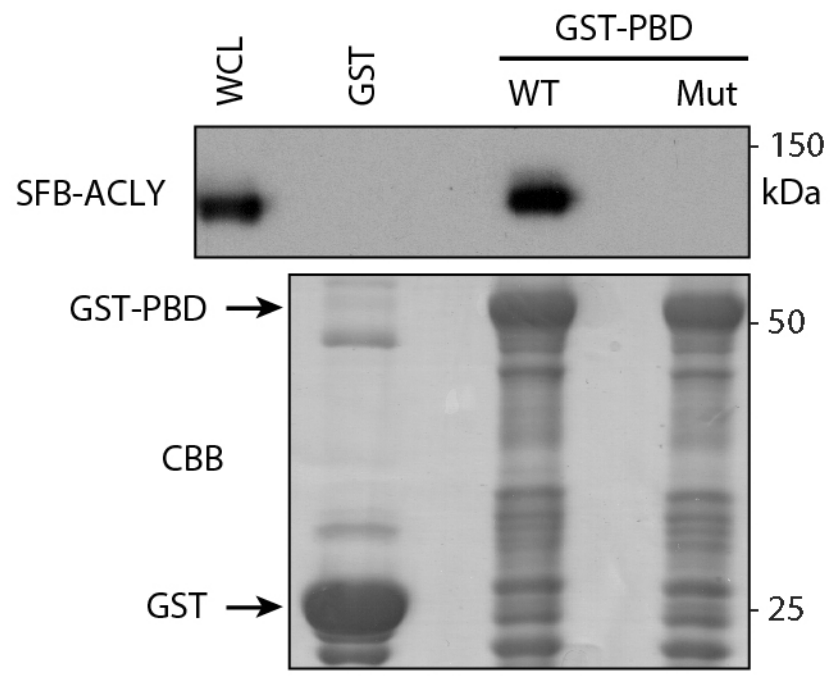

Fig. S1. Unbiased proteomics identifies ACLY as a phosphorylation-dependent Plk1 interactor. Related to Figure 1.

HEK293T cells expressing SFB-ACLY were subjected to pull-down with the indicated proteins, followed by CBB staining and immunoblotting with the antibody against the Flag tag.

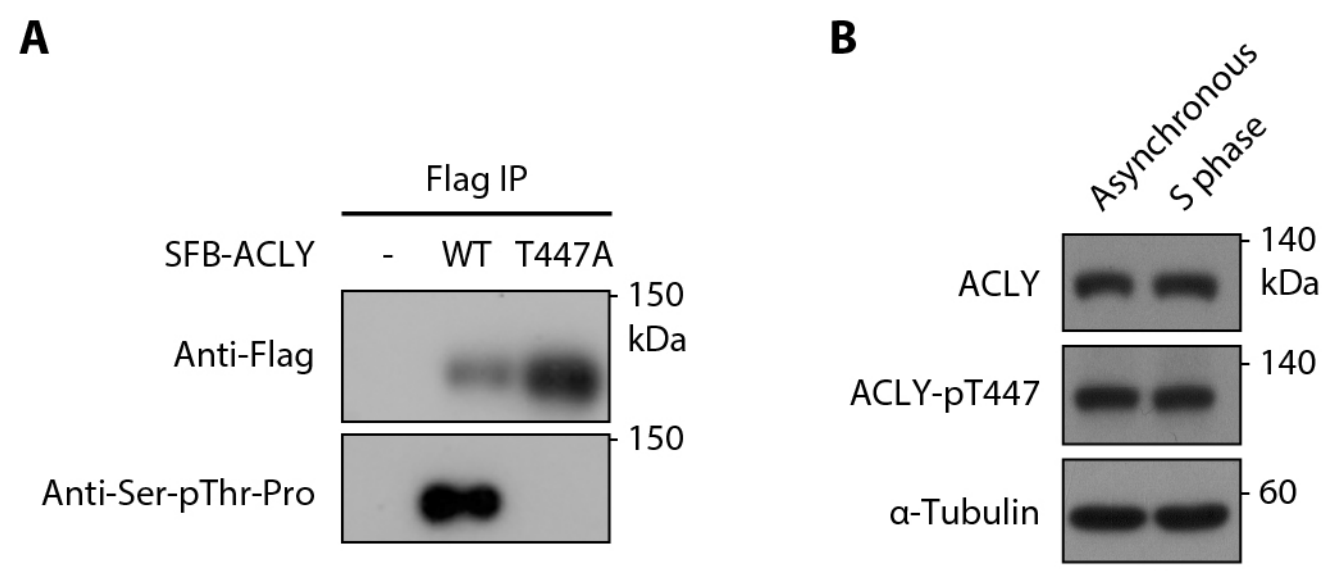

Fig. S2. Phosphorylation of ACLY at Thr-447 generates a docking site for Plk1. Related to Figure 2.

(**A**) HEK293T cells expressing SFB-ACLY (WT or T447A) or an empty vector control were subjected to immunoprecipitation with anti-Flag beads, followed by immunoblotting.

(**B**) HeLa cells were either left untreated or arrested in S phase by thymidine treatment for 24 hours, and then harvested and analyzed by immunoblotting.

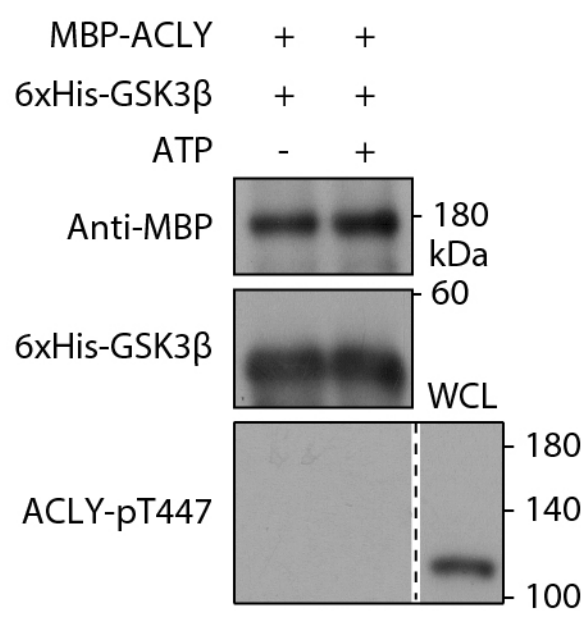

Fig. S3. GSK3β phosphorylates ACLY at T447 to prime Plk1 binding. Related to Figure 3.

MBP-ACLY was subjected to *in vitro* phosphorylation by 6xHis-GSK3β, followed by immunoblotting with the antibodies against MBP and GSK3β. The ACLY-pT447 signal in whole-cell lysates of HeLa cells served as a positive control. Irrelevant lanes were excised from the membrane image (indicated by a vertical line).

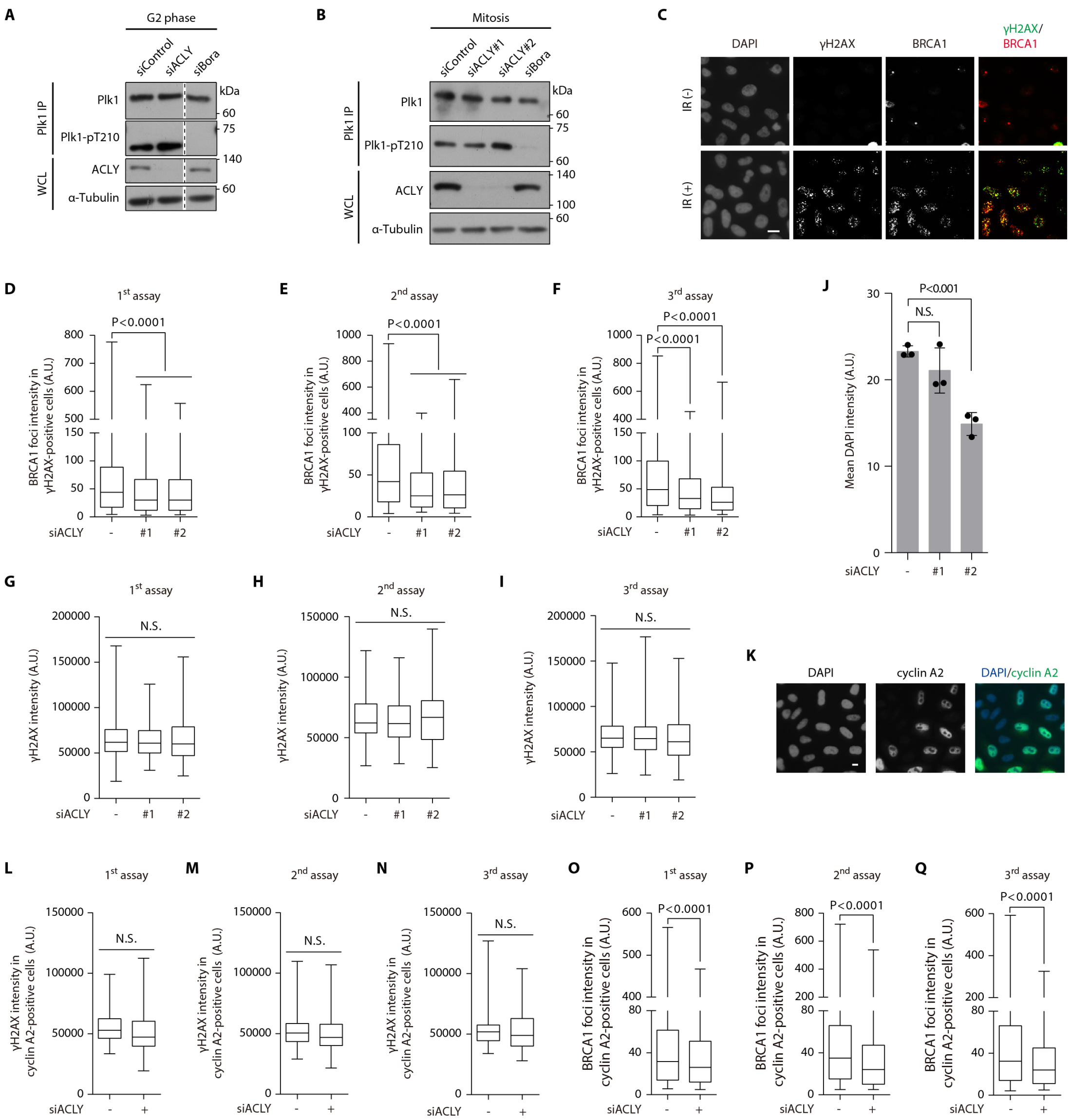

Fig. S4. ACLY promotes BRCA1 recruitment to DSBs without affecting Plk1 activity. Related to Figure 4.

(**A**) HeLa cells were transfected with the indicated siRNAs and synchronized using double-thymidine block. Eight hours after release, cells were subjected to immunoprecipitation using the Plk1 antibody, followed by immunoblotting. Irrelevant lanes were excised (indicated by a vertical line).

(**B**) HeLa cells were transfected with the indicated siRNAs. Thirty-two hours later, cells were treated with nocodazole for 16 h, and mitotic cells were collected for immunoprecipitation using the Plk1 antibody, followed by immunoblotting.

(**C**) HeLa cells were irradiated (2 Gy) or left untreated. Four hours later, cells were processed for immunofluorescence staining. Representative images are shown.

(**D** to **F**) HeLa cells were transfected with the indicated siRNAs. Forty-eight hours post-transfection, cells were irradiated (2 Gy) and processed for immunofluorescence

staining 4 hours later. Quantification of BRCA1 foci intensity in γH2AX-positive cells for each of the three individual experiments is shown (n >50 cells per condition). Boxplots depict the median, 25th and 75th percentiles, with whiskers indicating the minima and maxima. Related to Fig. 4D.

(**G** to **I**) HeLa cells were transfected with the indicated siRNAs. Forty-eight hours post-transfection, cells were irradiated (2 Gy) and processed for immunofluorescence staining 4 hours later. Quantification of nuclear γH2AX intensity for each of the three individual experiments is shown (n >100 cells per condition). Boxplots depict the median, 25th and 75th percentiles, with whiskers indicating the minima and maxima. Related to Fig. 4E.

(**J**) HeLa cells were transfected with the indicated siRNAs. Forty-eight hours post-transfection, cells were irradiated (2 Gy) and processed for immunofluorescence staining 4 hours later. Quantification of mean DAPI intensity is shown. Data are presented as mean ± SD from three independent experiments (n >300 cells per condition). Related to Fig. 4B.

(**K**) Asynchronously growing HeLa cells were irradiated (2 Gy) and processed for immunofluorescence staining 4 hours later. Representative images are shown.

(**L** to **N**) HeLa cells were transfected with the indicated siRNAs. Forty-eight hours post-transfection, cells were irradiated and processed for immunofluorescence staining 4 hours later. Quantification of nuclear γH2AX intensity in cyclin A2-positive cells for each of the three individual experiments is shown (n >100 cells per condition). Boxplots depict the median, 25th and 75th percentiles, with whiskers indicating the minima and maxima. Related to Fig. 4I.

(**O** to **Q**) HeLa cells were transfected with the indicated siRNAs. Forty-eight hours post-transfection, cells were irradiated and processed for immunofluorescence staining 4 hours later. Quantification of BRCA1 foci intensity in cyclin A2-positive cells for each of the three individual experiments is shown (n >50 cells per condition). Boxplots depict the median, 25th and 75th percentiles, with whiskers indicating the minima and maxima. Related to Fig. 4L.

Scale bars, 10 μm (C, K). A.U., arbitrary units (D-F, G-J, L-Q).

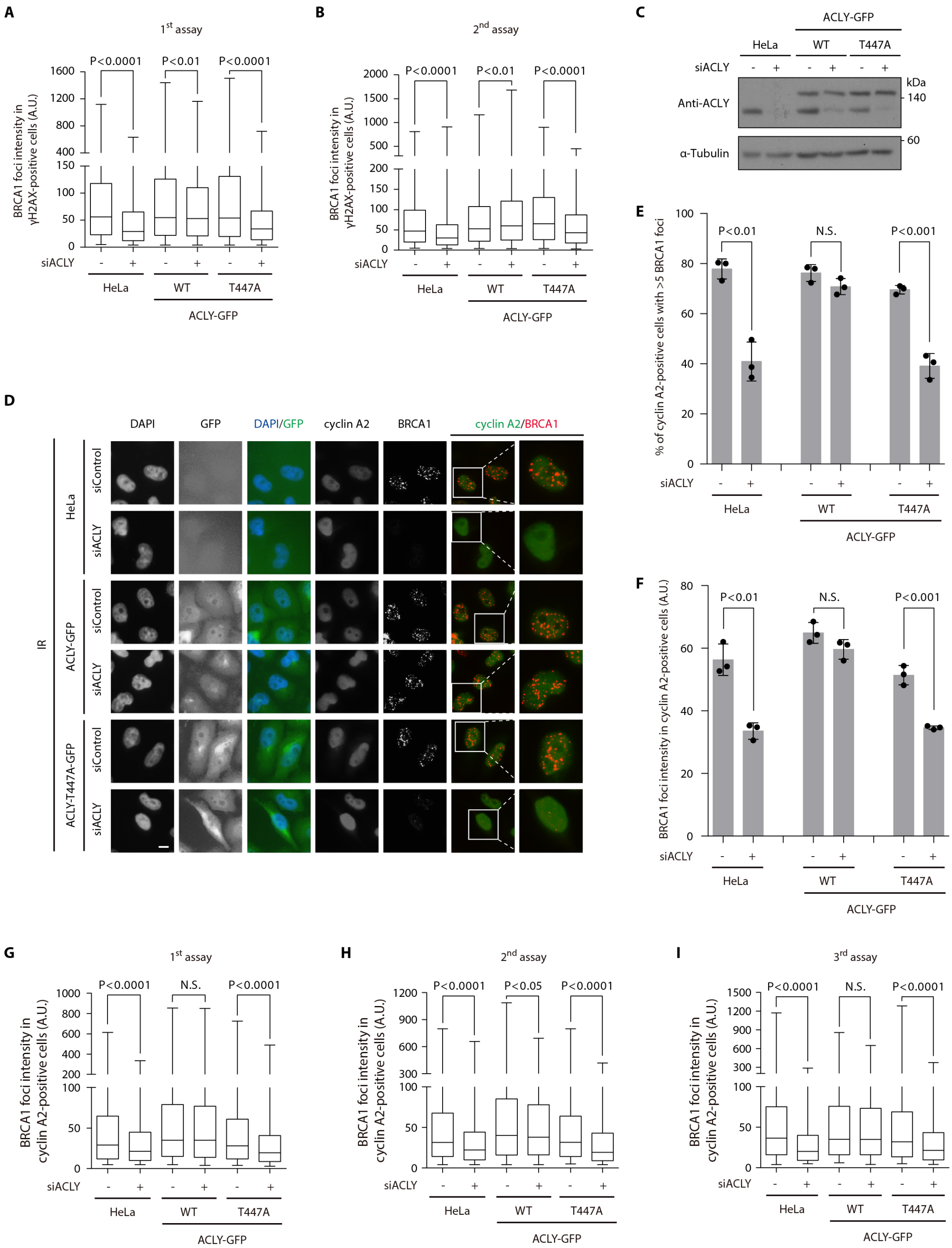

Fig. S5. GSK3β-mediated ACLY-T447 phosphorylation is required for efficient BRCA1 recruitment to DSBs. Related to Figure 5.

(**A** and **B**) Parental HeLa cells and HeLa cells stably expressing ACLY-GFP (WT or T447A) were transfected with control or ACLY siRNA. Two days post-transfection, cells were irradiated (2 Gy) and processed for immunofluorescence staining 4 hours later. Quantification of BRCA1 foci intensity in γH2AX-positive cells for each of the two individual experiments is shown (n >50 cells per condition). Boxplots depict the

median, 25th and 75th percentiles, with whiskers indicating the minima and maxima. Related to Figure 5E.

(**C** to **I**) Parental HeLa cells and HeLa cells stably expressing ACLY-GFP (WT or T447A) were transfected with control or ACLY siRNA. Two days post-transfection, cells were irradiated (2 Gy) and processed for immunoblotting (C) or immunofluorescence staining 4 hours later. Representative images are shown (D). Scale bars, 10 µm. Quantification of the percentage of γH2AX-positive cells containing >5 BRCA1 foci (E) and BRCA1 foci intensity in γH2AX-positive cells (F) is shown. Data are presented as mean ± SD from three independent experiments (n >300 cells per condition for E; n >150 cells per condition for F). Quantification of BRCA1 foci intensity in γH2AX-positive cells for each of the three individual experiments (G-I) is shown (n >50 cells per condition). Boxplots depict the median, 25th and 75th percentiles, with whiskers indicating the minima and maxima.

A.U., arbitrary units (D-F, G-J, L-Q).

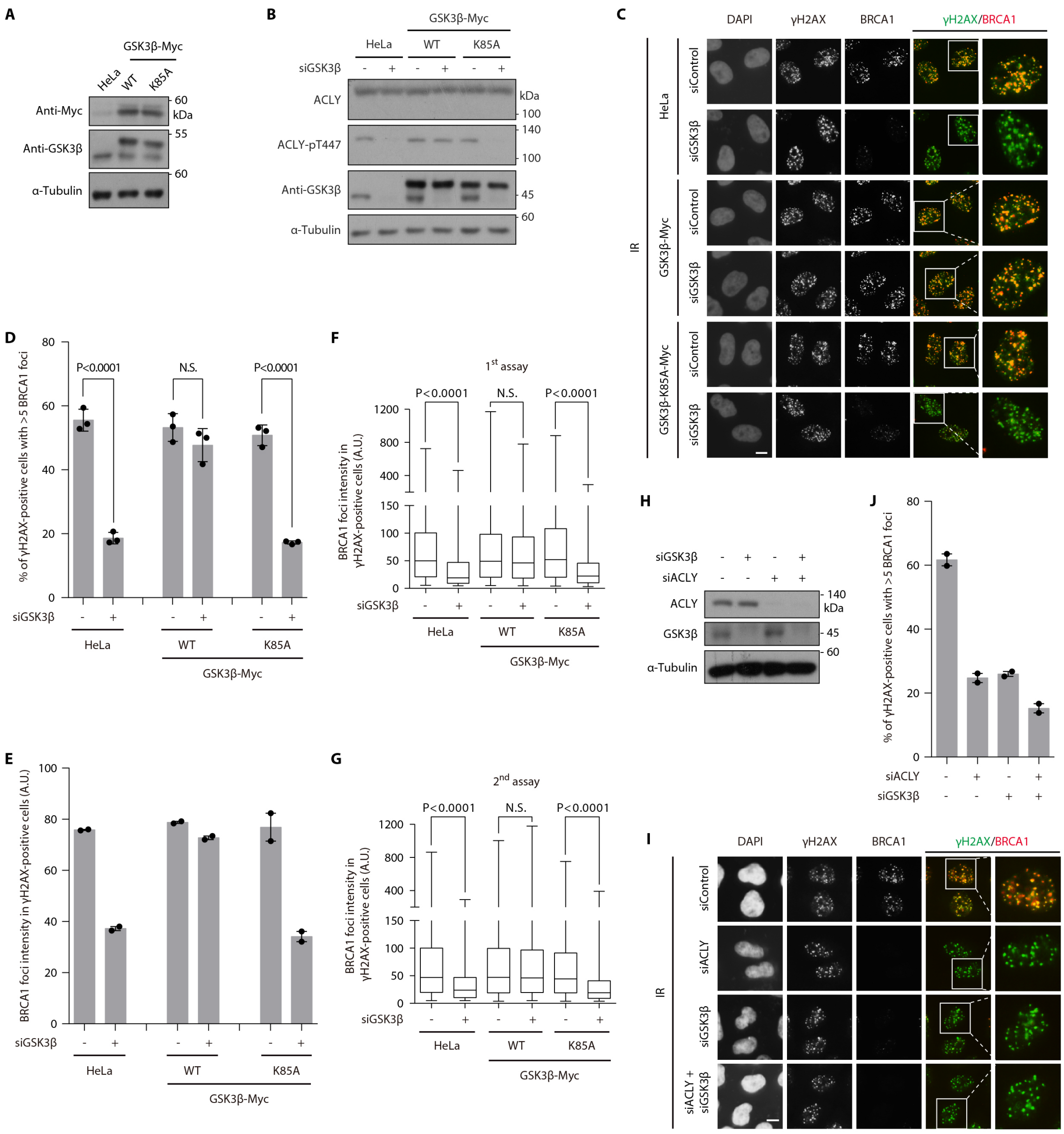

Fig. S6. GSK3β-mediated ACLY-T447 phosphorylation is required for efficient BRCA1 recruitment to DSBs. Related to Figure 5.

(**A**) Immunoblot analysis of whole-cell lysates from HeLa cells stably expressing GSK3β-Myc (WT or K85A).

(**B** to **G**) Parental HeLa cells and HeLa cells stably expressing GSK3β-Myc (WT or K85A) were transfected with control or GSK3β siRNA. Two days post-transfection, cells were irradiated (2 Gy) and processed for immunoblotting (B) or immunofluorescence staining 4 hours later. Representative images are shown (C). Quantification of the percentage of γH2AX-positive cells containing >5 BRCA1 foci is shown in (D). Data are presented as mean ± SD from three independent experiments (n > 300 cells per condition). Quantification of BRCA1 foci intensity in γH2AX-positive cells is shown in (E). Data are presented as mean and range from two

independent experiments (n > 100 cells per condition). Quantification of BRCA1 foci intensity in γH2AX-positive cells for each of the two individual experiments is also shown (F, G) (n > 50 cells per condition). Boxplots depict the median, 25th and 75th percentiles, with whiskers indicating minima and maxima. A.U., arbitrary units (D-F, G-J, L-Q).

(**H** to **J**) HeLa cells were transfected with the indicated siRNAs. Two days post-transfection, cells were irradiated (2 Gy) and processed for immunoblotting (H) or immunofluorescence staining 4 hours later. Representative images are shown (I). Quantification of the percentage of γH2AX-positive cells containing >5 BRCA1 foci is shown in (J). Data are presented as mean and range from two independent experiments (n > 200 cells per condition).

Scale bars, 10 μm (C, I).

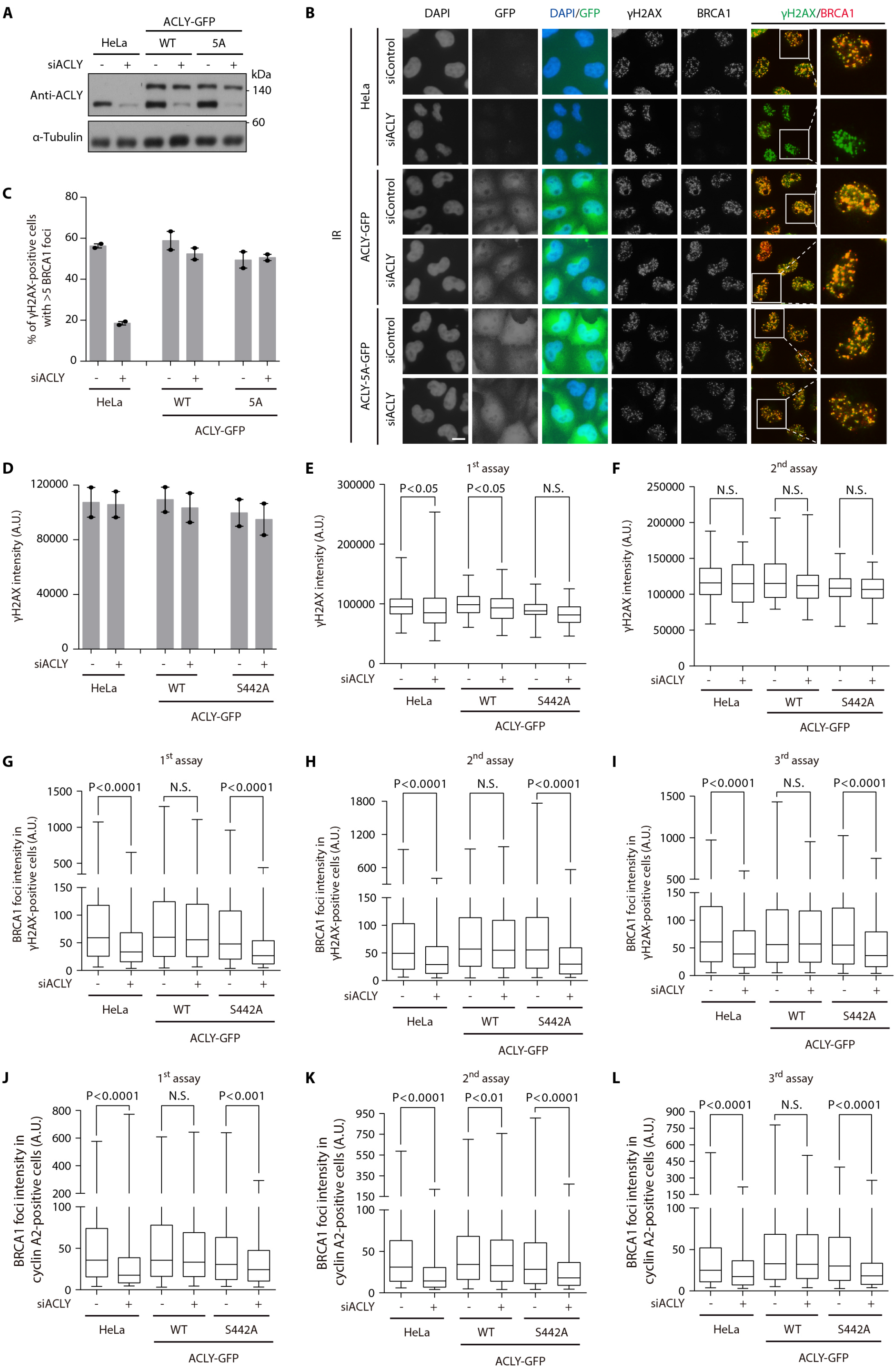
A
ACLY-GFP
HeLa
WT
5A
siACLY
- + - + - +
kDa
Anti-ACLY
140
60
α-Tubulin
B
DAPI
GFP
DAPI/GFP
γH2AX
BRCA1
γH2AX/BRCA1
IR
HeLa
siControl
siACLY
ACLY-GFP
siControl
siACLY
ACLY-5A-GFP
siControl
siACLY
C
% of γH2AX-positive cells with >5 BRCA1 foci
80
60
40
20
0
siACLY
- + - + - +
HeLa
WT
5A
ACLY-GFP
D
γH2AX intensity (A.U.)
120000
80000
40000
0
siACLY
- + - + - +
HeLa
WT
S442A
ACLY-GFP
E
1st assay
γH2AX intensity (A.U.)
300000
200000
100000
0
P<0.05
P<0.05
N.S.
siACLY
- + - + - +
HeLa
WT
S442A
ACLY-GFP
F
2nd assay
γH2AX intensity (A.U.)
250000
200000
150000
100000
50000
0
N.S.
N.S.
N.S.
siACLY
- + - + - +
HeLa
WT
S442A
ACLY-GFP
G
1st assay
BRCA1 foci intensity in γH2AX-positive cells (A.U.)
1500
1000
500
150
100
50
0
P<0.0001
N.S.
P<0.0001
siACLY
- + - + - +
HeLa
WT
S442A
ACLY-GFP
H
2nd assay
BRCA1 foci intensity in γH2AX-positive cells (A.U.)
1800
1200
600
150
100
50
0
P<0.0001
N.S.
P<0.0001
siACLY
- + - + - +
HeLa
WT
S442A
ACLY-GFP
I
3rd assay
BRCA1 foci intensity in γH2AX-positive cells (A.U.)
1500
1000
500
150
100
50
0
P<0.0001
N.S.
P<0.0001
siACLY
- + - + - +
HeLa
WT
S442A
ACLY-GFP
J
1st assay
BRCA1 foci intensity in cyclin A2-positive cells (A.U.)
800
600
400
200
100
50
0
P<0.0001
N.S.
P<0.001
siACLY
- + - + - +
HeLa
WT
S442A
ACLY-GFP
K
2nd assay
BRCA1 foci intensity in cyclin A2-positive cells (A.U.)
950
750
550
350
150
100
50
0
P<0.0001
P<0.01
P<0.0001
siACLY
- + - + - +
HeLa
WT
S442A
ACLY-GFP
L
3rd assay
BRCA1 foci intensity in cyclin A2-positive cells (A.U.)
900
750
600
450
300
150
100
50
0
P<0.0001
N.S.
P<0.0001
siACLY
- + - + - +
HeLa
WT
S442A
ACLY-GFP

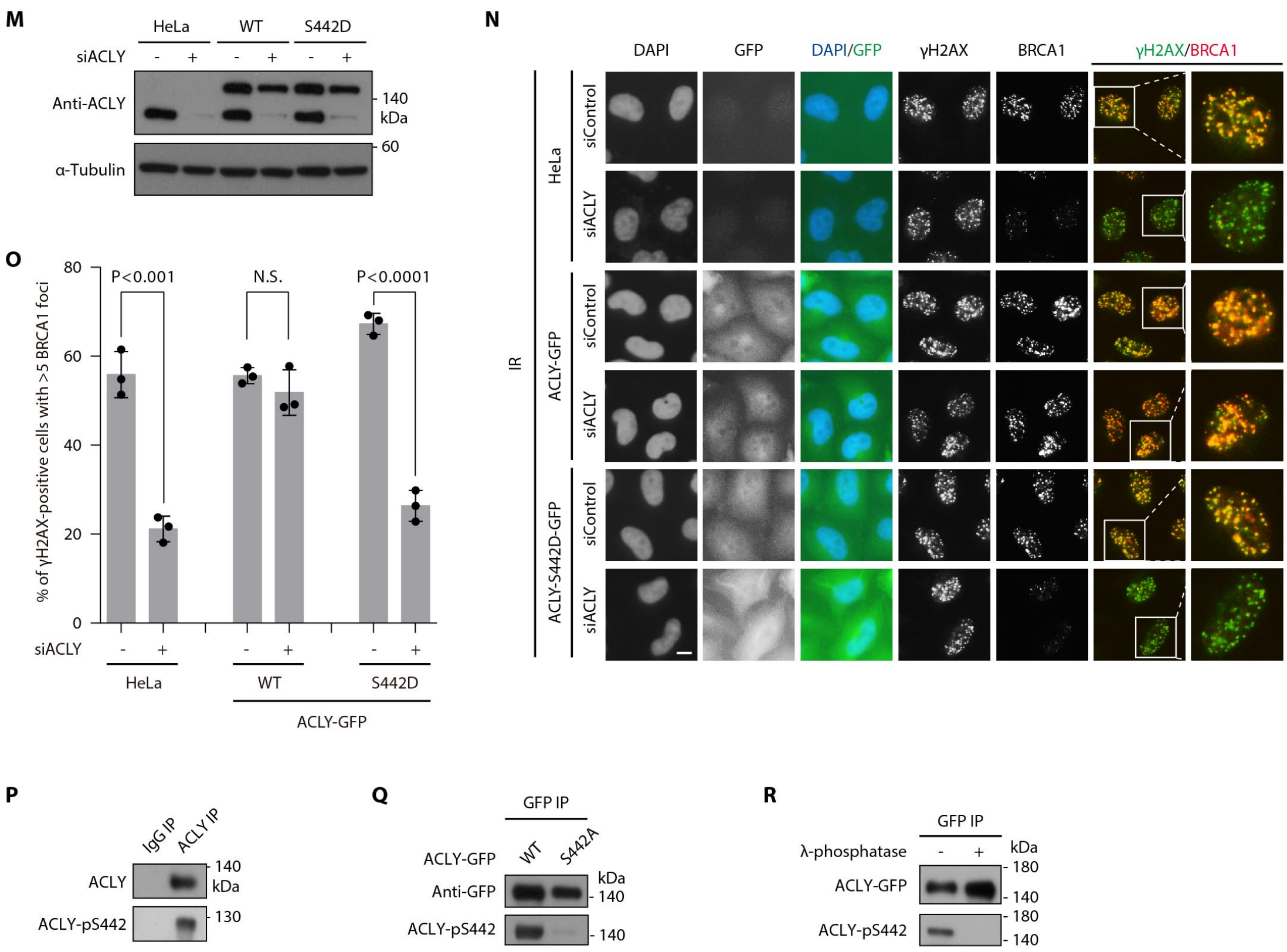

Fig. S7. Phosphorylation at Ser-442 is required for ACLY to promote BRCA1 accumulation at DSBs. Related to Figure 6.

(**A** to **C**) Parental HeLa cells and HeLa cells stably expressing ACLY-GFP (WT or 5A mutant) were transfected with control or ACLY siRNA. Forty-eight hours post-transfection, cells were irradiated (2 Gy) and processed for immunoblotting (A) or immunofluorescence staining 4 hours later. Representative images are shown (B). Quantification of the percentage of γH2AX-positive cells with >5 BRCA1 foci is shown (C). Data are presented as mean and range from two independent experiments (n >200 cells per condition).

(**D** to **F**) Parental HeLa cells and HeLa cells stably expressing ACLY-GFP (WT or S442A) were transfected with control or ACLY siRNA. Forty-eight hours post-transfection, cells were irradiated (2 Gy) and processed for immunofluorescence staining 4 hours later. The mean and range for γH2AX intensity from two independent experiments are shown (D) (n >100 cells per condition). Quantification of γH2AX intensity for each of the two individual experiments is shown (E, F) (n >50 cells per condition). Boxplots depict the median, 25th and 75th percentiles, with whiskers indicating minima and maxima. Related to Figure 6B.

(**G** to **I**) Parental HeLa cells and HeLa cells stably expressing ACLY-GFP (WT or S442A) were transfected with control or ACLY siRNA. Forty-eight hours post-transfection, cells were irradiated (2 Gy) and processed for immunofluorescence staining 4 hours later. Quantification of BRCA1 foci intensity in γH2AX-positive

cells for each of the three individual experiments is shown (n >50 cells per condition). Boxplots depict the median, 25th and 75th percentiles, with whiskers indicating minima and maxima. Related to Figure 6D.

(**J** to **L**) Parental HeLa cells and HeLa cells stably expressing ACLY-GFP (WT or S442A) were transfected with control or ACLY siRNA. Forty-eight hours post-transfection, cells were irradiated (2 Gy) and processed for immunofluorescence staining 4 hours later. Quantification of BRCA1 foci intensity in cyclin A2-positive cells for each of the three individual experiments is shown (n >50 cells per condition). Boxplots depict the median, 25th and 75th percentiles, with whiskers indicating minima and maxima. Related to Figure 6H.

(**M** to **O**) Parental HeLa cells and HeLa cells stably expressing siRNA-resistant ACLY-GFP (WT or S442D) were transfected with control or ACLY siRNA. Two days post-transfection, cells were irradiated (2 Gy) and processed for immunoblotting (M) or immunofluorescence staining 4 hours later. Representative images are shown (N). Quantification of the percentage of γH2AX-positive cells containing >5 BRCA1 foci is shown in (O). Data are presented as mean ± SD from three independent experiments (n > 300 cells per condition).

(**P**) HeLa cell lysates were subjected to immunoprecipitation using anti-ACLY antibody or control IgG, followed by immunoblotting.

(**Q**) HeLa cells stably expressing ACLY-GFP (WT or S442A) were subjected to immunoprecipitation with anti-GFP beads, followed by immunoblotting.

(**R**) Lysates of HeLa cells stably expressing ACLY-GFP were treated with λ-phosphatase or a vehicle control, followed by immunoprecipitation with anti-GFP beads and immunoblotting with the antibodies against GFP and ACLY-pS442.

Scale bars, 10 μm (B, N). A.U., arbitrary units (D-F, G-J, L-Q).

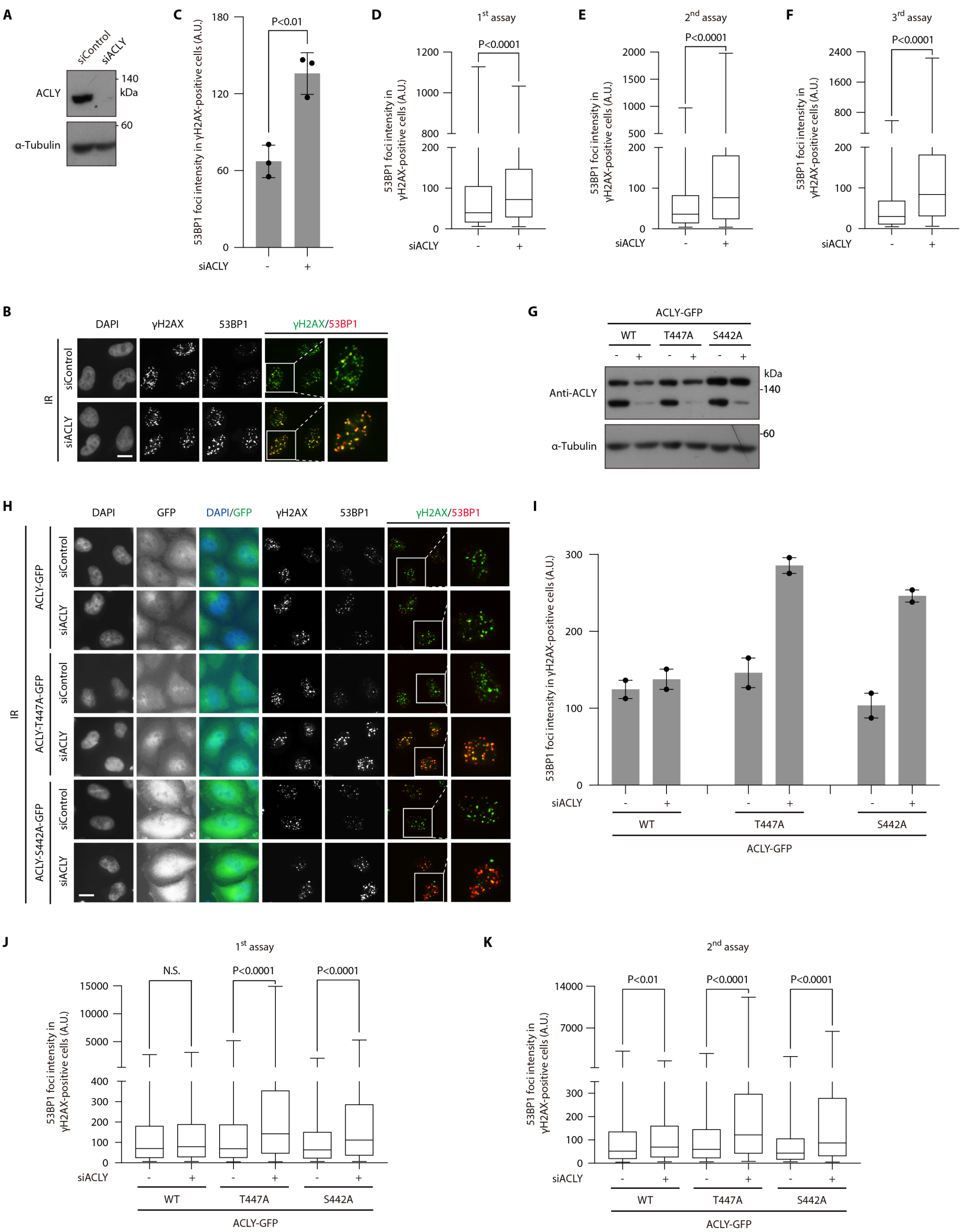

Fig. S8. Phosphorylation at Ser-442 is required for ACLY to promote BRCA1 accumulation at DSBs. Related to Figure 6.

(**A** to **F**) HeLa cells were transfected with control or ACLY siRNA. Forty-eight hours post-transfection, cells were irradiated (2 Gy) and processed for immunoblotting (A) or immunofluorescence staining 4 hours later. Representative images are shown (B). Quantification of 53BP1 foci intensity in γH2AX-positive cells is shown (C). Data are presented as mean ± SD from three independent experiments (n > 150 cells per condition). For 53BP1 intensity in γH2AX-positive cells in each of the three

individual experiments (D-F) (n >50 cells per condition), boxplots depict the median, 25th and 75th percentiles, with whiskers indicating the minima and maxima.

(**G** to **K**) HeLa cells stably expressing ACLY-GFP (WT, T447A, or S442A) were transfected with control or ACLY siRNA. Forty-eight hours post-transfection, cells were irradiated (2 Gy) and processed for immunoblotting (G) or immunofluorescence staining 4 hours later. Representative images are shown (H). Quantification of 53BP1 foci intensity in γH2AX-positive cells is presented (I). Data are shown as mean and range from two independent experiments (n > 100 cells per condition). For 53BP1 intensity in γH2AX-positive cells in each of the two individual experiments (J, K) (n >50 cells per condition), boxplots depict the median, 25th and 75th percentiles, with whiskers indicating the minima and maxima.

Scale bar, 10 µm (B, H). A.U., arbitrary units (D-F, G-J, L-Q).

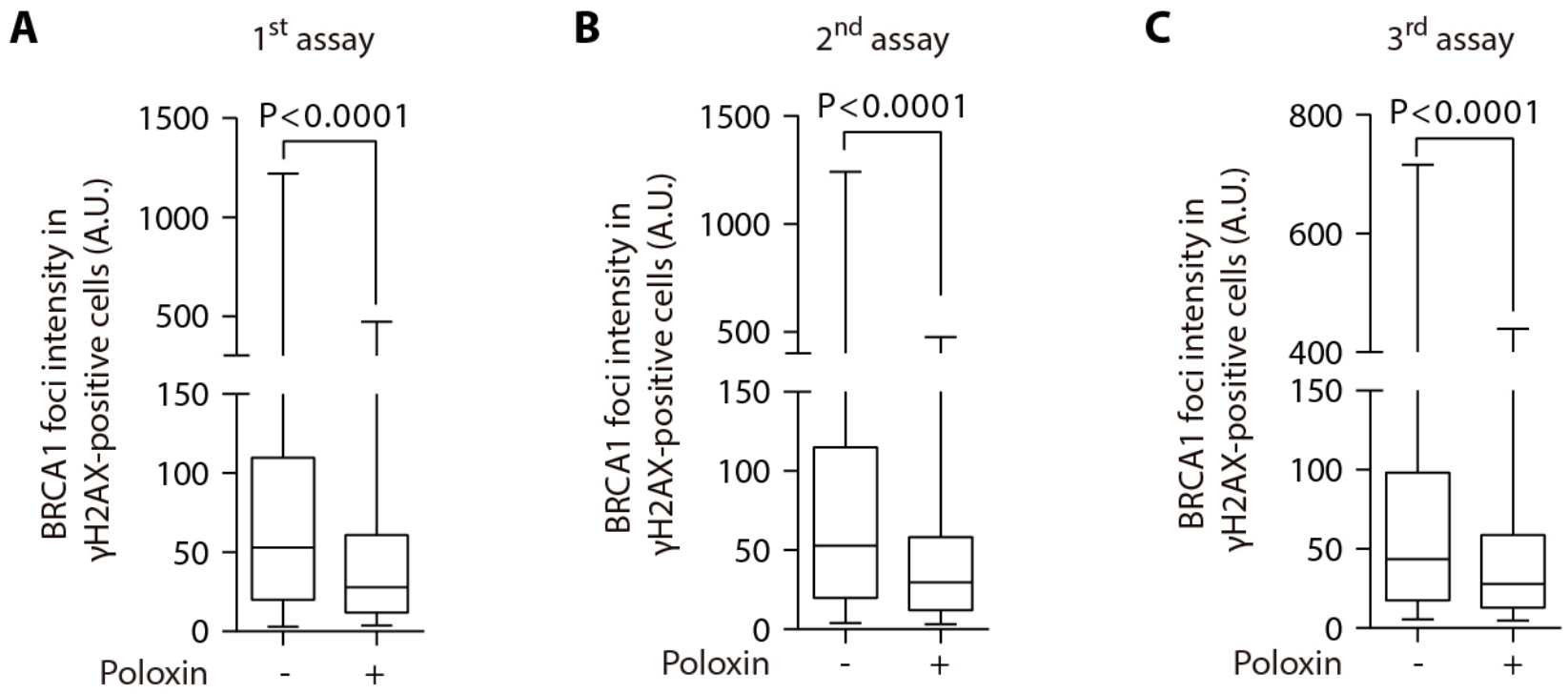

Fig. S9. Plk1 phosphorylates ACLY at Ser-442 to promote BRCA1 accumulation at DSBs. Related to Figure 7.

(**A** to **C**) HeLa cells treated with Poloxin for 3 hours were irradiated (2 Gy) and processed for immunofluorescence staining 4 hours later. Quantification of BRCA1 foci intensity in γH2AX-positive cells for each of the three individual experiments is shown (n >50 cells per condition). Boxplots depict the median, 25th and 75th percentiles, with whiskers indicating the minima and maxima. A.U., arbitrary units (D-F, G-J, L-Q). Related to Figure 7H.

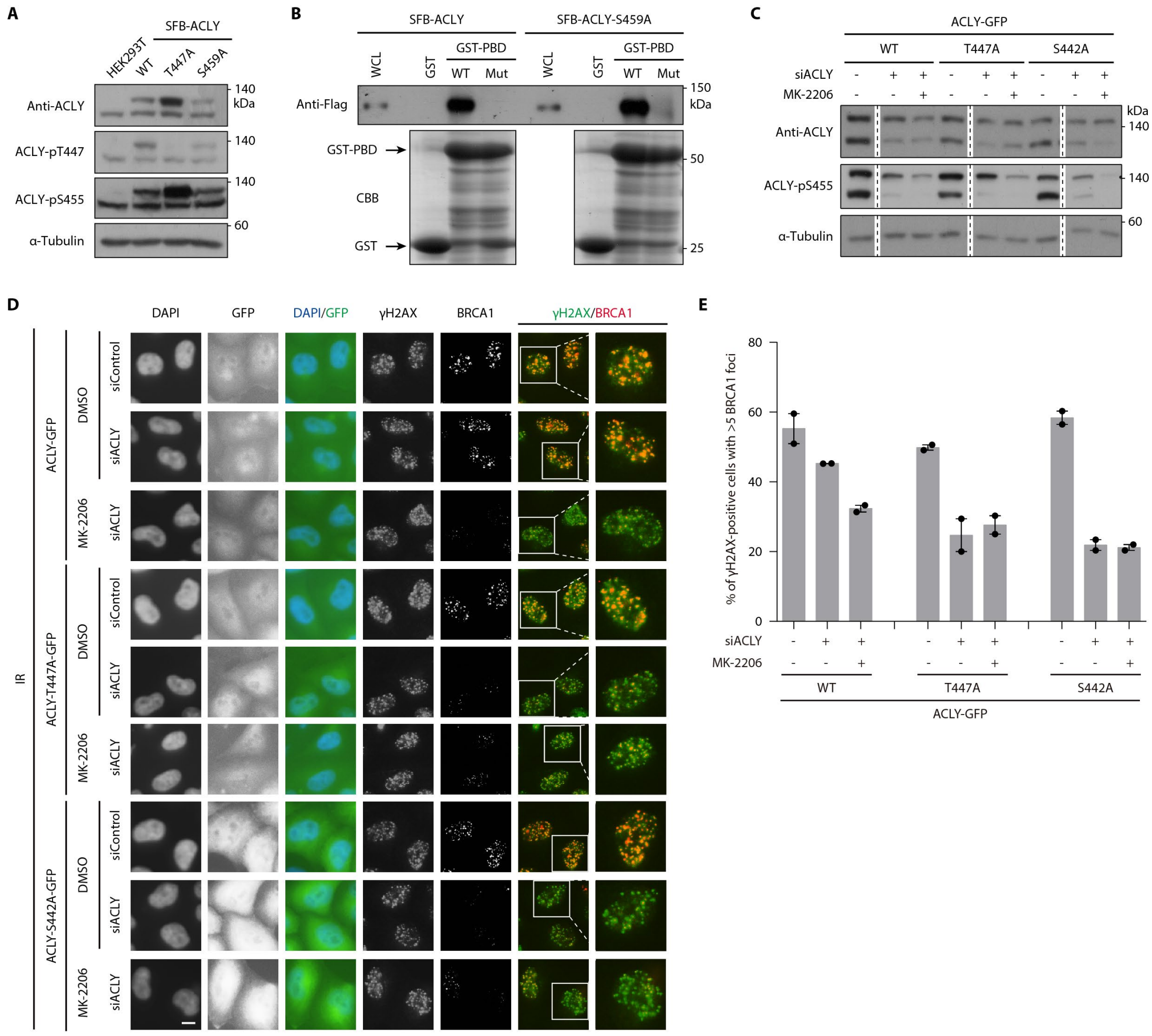

Fig. S10. AKT-dependent phosphorylation of ACLY at Ser-455 primes Thr-447 phosphorylation by GSK3β. Related to Figure 8.

(**A**) HEK293T cells expressing SBF-ACLY (WT, T447A or S459A), or an empty vector control, were analyzed by immunoblotting.

(**B**) HEK293T cells expressing SBF-ACLY (WT or S459A) were subjected to pull-down with the indicated proteins, followed by immunoblotting and CBB staining.

(**C** to **E**) HeLa cells stably expressing ACLY-GFP (WT, T447A, or S442A) were transfected with the indicated siRNAs. Forty-eight hours post-transfection, cells were treated with DMSO (control) or MK-2206 for 5 hours and then irradiated (2 Gy) and processed for immunoblotting (C) or immunofluorescence staining 4 hours later. Irrelevant lanes were excise (indicated by a vertical line). Representative images are shown (D). Scale bars, 10 μm. Quantification of the percentage of γH2AX-positive cells with >5 BRCA1 foci is shown (E). Data are presented as mean and range from two independent experiments (n >100 cells per condition).

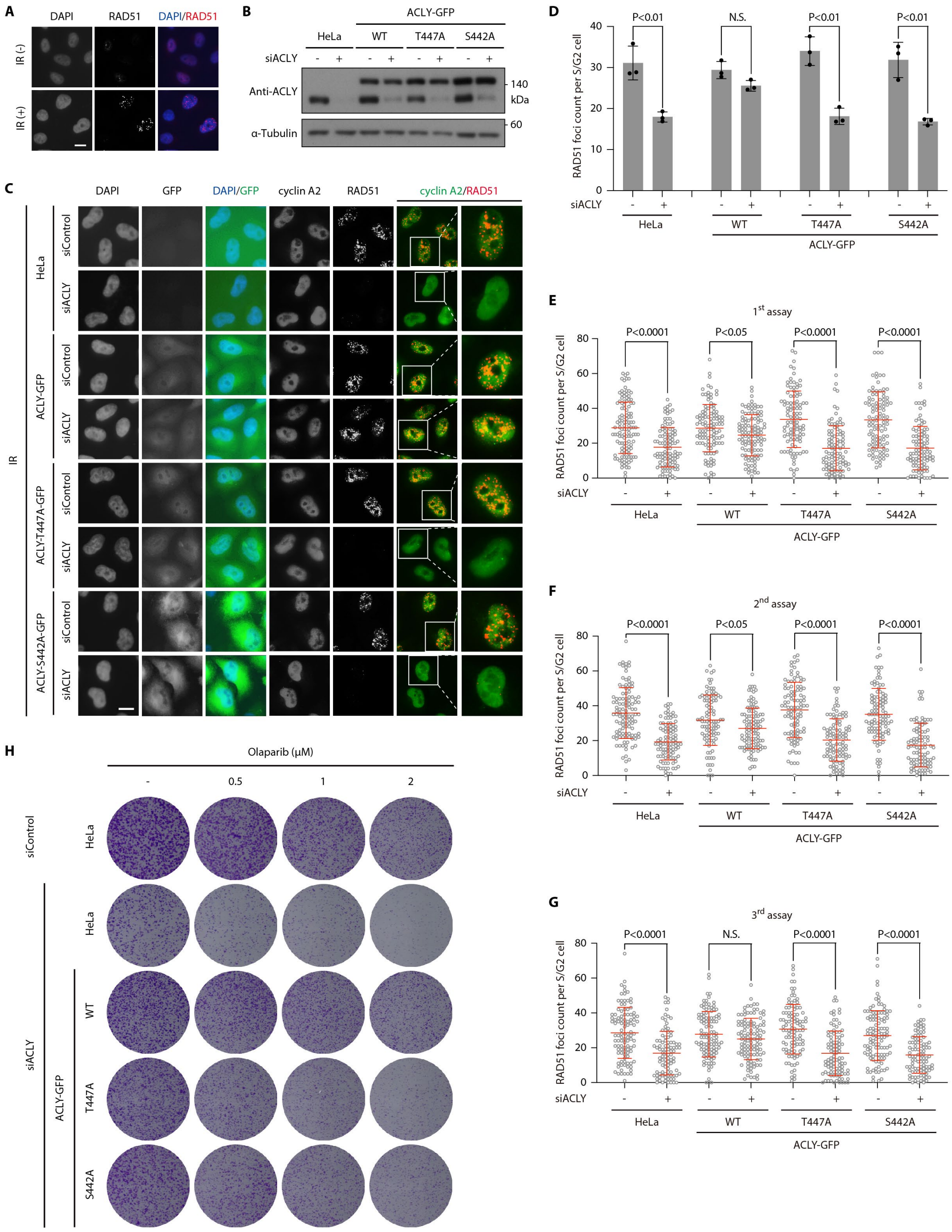

Fig. S11. Plk1-mediated ACLY-S442 phosphorylation promotes RAD51 loading at DSBs and confers resistance to PARP inhibition. Related to Figure 9.

(**A**) HeLa cells were either irradiated (4 Gy) or left untreated. Four hours later, cells were processed for immunofluorescence staining.

(**B** to **G**) Parental HeLa cells and HeLa cells stably expressing ACLY-GFP (WT, T447A, or S442A) were transfected with control or ACLY siRNA. Forty-eight hours later, cells were irradiated (4 Gy) and processed for immunoblotting (B) or

immunofluorescence staining 4 hours later. Representative images are shown in (C). Quantification of RAD51 foci counts in cyclin A2-positive cells is shown in (D). Data are presented as mean ± SD from three independent experiments (n >150 cells per condition). Quantification of RAD51 foci counts for each of the three individual experiments is shown (E-G).

(**H**) The indicated cell lines were transfected with control or ACLY siRNA, treated with olaparib, and subjected to clonogenic survival assays as in Figure 9G. Representative images of crystal violet-stained colonies are shown. Quantification of surviving fractions is presented in Figure 9G.

Scale bar, 10 µm (A, C).

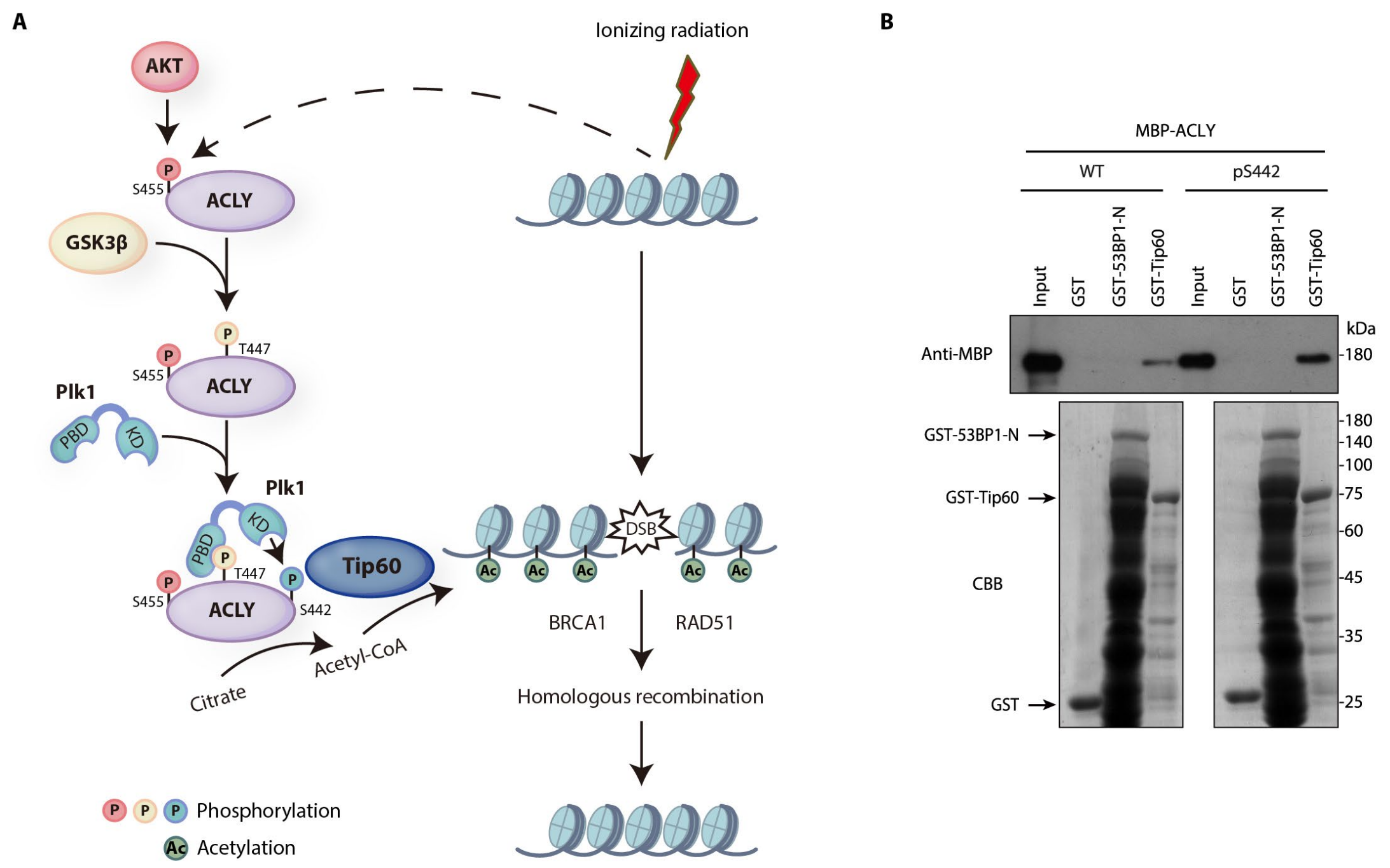

Fig. S12. Plk1-mediated phosphorylation of ACLY-S442 enhances the ACLY-Tip60 interaction and sustains histone H4 acetylation. Related to Figure 10.

(**A**) Schematic model illustrating how sequential phosphorylation of ACLY by AKT, GSK3β and Plk1 sustains histone acetylation to promote HR.

(**B**) Recombinant MBP-ACLY (WT or pS442) was subjected to pull-down with GST-Tip60, GST-53BP1-N, or GST alone, followed by immunoblotting and CBB staining.

Data S1 (separate file in excel format; Related to Fig. 1B): Mass spectrometry analysis of proteins pulled down by GST from HeLa cell lysates.

Data S2 (separate file in excel format; Related to Fig. 1B): Mass spectrometry analysis of proteins pulled down by GST-PBD from HeLa cell lysates.

Data S3 (separate file in excel format; Related to Fig. 1B): Mass spectrometry analysis of proteins pulled down by GST-PBDmut from HeLa cell lysates.
